# The impacts of warming and hypoxia on the performance of an obligate ram ventilator

**DOI:** 10.1093/conphys/coz026

**Published:** 2019-06-19

**Authors:** Daniel P Crear, Rich W Brill, Peter G Bushnell, Robert J Latour, Gail D Schwieterman, Rachel M Steffen, Kevin C Weng

**Affiliations:** 1 Fisheries Science Department, Virginia Institute of Marine Science, William & Mary, Gloucester Point, VA, USA; 2 Department of Biological Sciences, Indiana University South Bend, South Bend, IN, USA; 3 Biology Department, Washington & Lee University, Lexington, VA, USA

**Keywords:** Accelerometer, aerobic scope, critical oxygen concentration, climate change, respirometry

## Abstract

Climate change is causing the warming and deoxygenation of coastal habitats like Chesapeake Bay that serve as important nursery habitats for many marine fish species. As conditions continue to change, it is important to understand how these changes impact individual species’ behavioral and metabolic performance. The sandbar shark (*Carcharhinus plumbeus*) is an obligate ram-ventilating apex predator whose juveniles use Chesapeake Bay as a nursery ground up to 10 years of age. The objective of this study was to measure juvenile sandbar shark metabolic and behavioral performance as a proxy for overall performance (i.e. fitness or success) when exposed to warm and hypoxic water. Juvenile sandbar sharks (79.5–113.5 cm total length) were collected from an estuary along the eastern shore of Virginia and returned to lab where they were fitted with an accelerometer, placed in a respirometer and exposed to varying temperatures and oxygen levels. Juvenile sandbar shark overall performance declined substantially at 32°C or when dissolved oxygen concentration was reduced below 3.5 mg l^−1^ (51% oxygen saturation between 24–32°C). As the extent of warm hypoxic water increases in Chesapeake Bay, we expect that the available sandbar shark nursery habitat will be reduced, which may negatively impact the population of sandbar sharks in the western Atlantic as well as the overall health of the ecosystem within Chesapeake Bay.

## Introduction

Climate change is warming coastal areas and estuaries worldwide. An increase in anthropogenic nutrient input is likewise increasing the severity and extent of hypoxic episodes in many of these areas (Hagy *et al.*, 2004; Preston, 2004; Kemp *et al.*, 2005; Conley *et al.*, 2007; Rabalais *et al.*, 2009; Najjar *et al.*, 2010; Irby *et al.*, 2016). These conditions are expected to worsen over the next 100 years as climate change impacts are exacerbated (Rabalais *et al.*, 2009; Najjar *et al.*, 2010; Irby *et al.*, 2018).

Changing environmental conditions in coastal areas and estuaries are likely to impact marine fish species that rely on these habitats as primary nursery grounds (Rijnsdorp *et al.*, 2009). For example, while in their nursery habitat, juvenile weakfish (*Cynoscion regalis*) avoided waters in a tributary of Indian River Bay, DE, USA with dissolved oxygen below 2 mg l^−1^ (Tyler and Targett, 2007). The prevalence of juvenile bull sharks (*Carcharhinus leucas*) has actually increased in their nursery ground habitat within Pamlico Sound, NC, USA over the last decade as a result of increased water temperatures (Bangley *et al.*, 2018). Shifts in species distribution, similar to the examples above, can lead to changes in the timing of migration of juveniles and adults (Nye *et al.*, 2009; Turner *et al.*, 2015), reproductive patterns of adults (Last *et al.*, 2011) and abundance of all life stages (Last *et al.*, 2011; Lynch *et al.*, 2014); all of which can influence population level success and the overall health of ecosystems (Morley *et al.*, 2018).

To understand these potential changes in fish ecology, it is important to assess the relationship between environmental conditions and the performance of individual fish. Performance is often assessed through measurement of aerobic scope [AS, i.e. the difference between maximum and standard metabolic rates (SMRs); Claireaux and Lefrançois, 2007; Di Santo, 2016], which quantifies an individual’s metabolic power (i.e. energy use per unit time) under which all of life’s processes beyond basic maintenance (e.g. growth, reproduction, digestion and movement) must occur (Clark *et al.*, 2013). Consistent with the theory of oxygen- and capacity-limited thermal tolerance (OCLTT), AS is a measure of fitness and performance in ectotherms. According to OCLTT, AS follows a bell-shaped curve with temperature such that there is a temperature where AS is optimized (Fry, 1971; Clark *et al.*, 2013). Therefore, it is expected that long-term warming will reduce AS and decrease the ability for many individuals to carry out multiple life processes simultaneously unless individuals adjust their range and distribution (Pörtner and Knust, 2007). However, the theory of OCLTT does not hold true for all species, as some demonstrate an increase in AS with temperature until temperature approaches lethal levels (Clark *et al.*, 2013; McKenzie *et al.*, 2016). This suggests that, for some species, the temperature at which AS is optimized is not equivalent to the temperature at which performance is maximized (Lefevre, 2016).

Increases in the extent and severity of hypoxic episodes in coastal areas are affecting the physiology and thus ecology of coastal species (Diaz and Rosenberg, 1995; Wannamaker and Rice, 2000; Tyler and Targett, 2007; Ludsin *et al.*, 2009; Zhang *et al.*, 2009). Hypoxia tolerance is often quantified by measurement of critical oxygen levels or oxygen level at which individuals can no longer maintain SMR. The critical oxygen saturation (S_crit_) should increase with temperature due to increases in SMR and decreases in oxygen solubility in water. Individuals cannot occupy waters long term with an oxygen level below S_crit_ because, under these conditions, at least some of the power needed to maintain homeostasis must be met through anaerobic metabolism (Fry and Hart, 1948; Schurmann and Steffensen, 1997; Brill *et al.*, 2015). Similar to S_crit_, the quantities C_crit_ and P_crit_ are the concentration and partial pressure of oxygen, respectively, at which a fish can no longer maintain SMR. All of these measures of critical oxygen, as well as AS, can be determined through intermittent-flow respirometry (Clark *et al.*, 2011; Lapointe *et al.*, 2014).

Measuring metabolic performance (i.e. AS) of an obligate ram ventilator, like a tuna or some shark species, is difficult because of the necessity of individuals to swim constantly and, therefore, a need to measure activity simultaneously. This requires either a swim tunnel respirometer where swimming speed can be controlled or a respirometer large enough for fish to swim independently coupled with a method to quantify activity. Since many species exhibit difficulty swimming in a swim tunnel, a large circular tank can be used as the respirometer such that fish are able to swim freely (Lear *et al.*, 2017). Because the swimming behavior and activity of fish cannot be controlled in the large circular respirometer accelerometers have been used to measure activity during experimentation (Lear *et al.*, 2017). Activity measurements (i.e. behavioral performance), obtained through accelerometers (Whitney *et al.*, 2007; Whitney *et al.*, 2016), can be used as a performance metric to quantify mechanical work, be used to describe behavior (Gleiss *et al.*, 2011; Payne *et al.*, 2018) and be used as an indicator to understand locomotor performance under different environmental regimes (Payne *et al.*, 2016; Payne *et al.*, 2018). Activity is correlated with metabolic performance, which suggests that accelerometers can be used to infer field metabolic rate when applied to free-ranging individuals (Bouyoucos *et al.*, 2017; Lear *et al.*, 2017). However, the relationship between accelerometer-derived activity metrics and metabolic rate has not been assessed under high levels of environmental stress.

The sandbar shark (*Carcharhinus plumbeus*) is an obligate ram-ventilating species that relies on coastal habitats as nursery grounds during younger life stages. In late spring, pupping occurs in Chesapeake Bay and coastal estuaries along the mid-Atlantic, where young-of-year remain through summer (Conrath and Musick, 2007; Grubbs and Musick, 2007). After moving south or offshore during winter, juveniles return to these nursery areas to forage and avoid larger predators during summer for the following 4–10 years (Grubbs and Musick, 2007). As a result of climate change and anthropogenic nutrient input, Chesapeake Bay is becoming warmer and more hypoxic (Hagy *et al.*, 2004; Preston, 2004; Kemp *et al.*, 2005; Najjar *et al.*, 2010). Temperature and oxygen limitations are not well understood for juvenile sandbar sharks. Therefore, the objective of this study was to measure juvenile sandbar shark metabolic and behavioral performance as a proxy of overall performance (i.e. fitness or success) when exposed to warm and hypoxic water.

## Materials and methods

### Shark collection and maintenance

A total of 13 juvenile sandbar sharks [79.5–113.5 cm total length (TL); 2.6–7.8 kg] were collected along the eastern shore of Virginia and brought back to the Virginia Institute of Marine Science Eastern Shore Lab, Wachapreague, VA, USA during the summers of 2016 and 2017 (Table 1). Individuals were held in a fiberglass tank (6.1 m diameter, 45 000 L) with a flow-through seawater system and acclimated to captivity for at least 2 weeks prior to experimentation. Sharks were fed frozen menhaden (*Brevoortia tyrannus*) until satiation every 3–5 days. All protocols for shark sampling, handling and experimentation were approved by the College of William and Mary Institutional Animal Care and Use Committee (protocol no. IACUC-2017-05-26-12 133-kcweng).

**Table 1:** Size and treatment summary of the juvenile sandbar sharks in the study

Animal ID	Year tested	Animal mass (kg)	TL (cm)	Treatments (°C)	Accelerometer
SB02	2016	7.30	109	24, 28, 32	No
SB03	2016	2.66	80	24, 28, 32	No
SB04	2016	5.09	99	24, 28, 32	No
SB05	2016	5.46	98	24, 28, 32	No
SB12	2016	5.69	103.5	24, 28, 32	No
SB28	2017	7.76	113.5	24, 28, 32	Yes
SB29	2017	3.31	91	24, 28, 32	Yes
SB30	2017	4.22	94	24, 28, 32	Yes
SB31	2017	3.98	91	24, 28, 32	Yes
SB32	2017	2.56	81	24, 28	Yes
SB33	2017	6.81	109	24, 28, 32	Yes
SB34	2017	6.84	107	24, 28, 32	Yes
SB37	2017	2.60	79.5	32	Yes

### Experimental design

**Figure 1:**
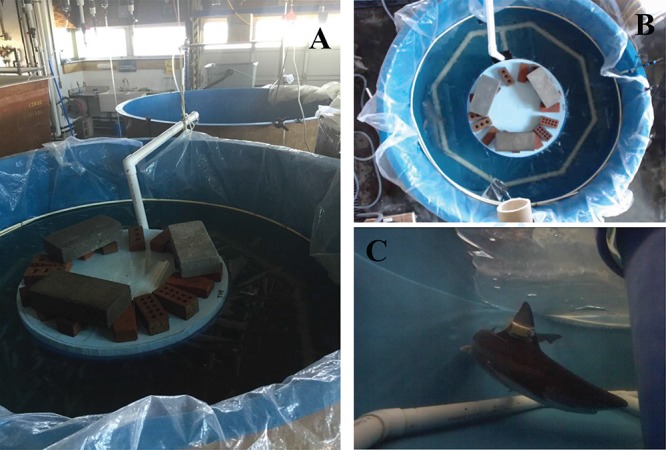
(**A**) The sandbar shark respirometer system with the respirometer in the foreground and the reservoir in the background. A PVC pipe connects the flush pump to the diffuser in the bottom of the respirometer. (**B**) An overhead view of the respirometer with a shark present. The PVC octagon is the diffuser that ensures mixing during the flush cycle. (**C**) A shark in the respirometer with an accelerometer fitted to its dorsal fin.

Maximum metabolic rate (MMR), minimum routine metabolic rate (mRMR; a proxy for SMR because sandbar sharks are obligate ram ventilators), AS and S_crit_ were measured in 12 sharks at 24°C, 28°C and 32°C using intermittent-flow respirometry to understand the effects of warming and hypoxia on sandbar shark physiology (Lapointe *et al.*, 2014; Brill *et al.*, 2015). Eleven sharks were tested at all three treatments (24, 28, and 32°C), while data collected at 24°C and 28°C from one shark was combined with data collected at 32°C from a different shark to represent one set of temperature treatments due to a mortality suffered in the holding tank between experiments. Sharks were acclimated to treatment temperatures in their holding tank through natural increases in water temperature throughout the summer (range: 18.0–33.8°C) because we were unable to control temperature in our holding tank. However, by using this approach, we were able to maximize ecological relevance. When experimental temperatures exceeded holding tank temperatures, individuals were transferred to a separate tank at the experimental temperature for at least 24–48 h prior to being introduced into the respirometer. The respirometry system consisted of two fiberglass tanks (2.4 m diameter, 1500 L volume) such that the first served as the respirometer and the second was a water reservoir used to flush the respirometer. To reduce the volume of the respirometer and ensure each experimental shark would swim in a circle, a negatively buoyant, smaller tank was placed in the center of the respirometer to create a circular track. A clear plastic sheet was placed over the respirometer and cinched to the side of the tank to prevent air–water gas exchange. Temperature was controlled by a large chiller unit with the addition of a heater kit (Teco TK 6000 Aquarium Chiller, Aquatic Solutions), while oxygen levels were controlled by bubbling air (normoxia) or nitrogen gas (hypoxia) into the water. The two tanks were connected through a drain on the bottom and PVC tubing over the top (Fig. 1A and B). Oxygen levels in the respirometer and reservoir were monitored every second using a temperature compensated, two-channel FireSting oxygen meter (Pyroscience) equipped with fiber optic oxygen probes that were fixed along the side of the respirometer and reservoir. Output from the oxygen probe in the respirometer was recorded in the FireStingO_2_ software and relayed to a program designed in Dasylab 9.02 (National Instruments) specifically for repeatedly recording metabolic rate measurements in intermittent-flow respirometry (Lapointe *et al.*, 2014; Brill *et al.*, 2015). The program also controlled the flow of nitrogen through a solenoid valve to maintain precise oxygen levels using output from the oxygen probe in the reservoir.

During a trial, the respirometry system cycled between the flushing and measurement period. During the flushing period (ranged from 30–45 min), oxygen and temperature-controlled water was pumped up and out of the reservoir, into the respirometer, and through a diffuser at the bottom of the respirometer (Fig. 1B) to ensure thorough mixing. Water flowed back into the reservoir through the bottom drain between the two tanks, allowing a continuous exchange of water between the two tanks during the flushing period. During the 15-min measurement period, the flush pump was turned off, and the shark’s oxygen consumption reduced the oxygen in the respirometer. We assumed the shark’s swimming motion adequately mixed the water in the respirometer during trials. The measurement period consisted of a 3-min equilibration interval (to ensure oxygen mixing in the respirometer) followed by 12 min of data recording to measure the rate of oxygen decline. The slope of a linear regression model fitted to the oxygen measurements was used to calculate metabolic rate using the equation

$$ {MO}_2=b\ x\ V\ x\ {W}^{-1}, $$

where *MO_2_* = metabolic rate (mg O_2_ kg^−1^h^−1^), $b$ = rate of change of oxygen content (estimated slope of linear regression) over the 12-min recording period (s^−1^), *V* = respirometer volume (l) corrected for the volume of the shark and *W* = weight of the shark (kg). Metabolic rate measurements where the regression *R*^2^ value was below 0.8 were eliminated and assumed to be compromised due to either poorly mixed water in the respirometer or contact between the shark and oxygen probe (which did occur occasionally).

To account for microbial respiration, oxygen consumption measurements were made in the absence of an experimental shark for at least 3 h prior to, and after, each trial. Based on those oxygen consumptions, a linear regression was used to estimate the rate of oxygen decline due to microbial respiration during the trial. The estimated oxygen consumptions were then subtracted from the measured rates of oxygen decline when the shark was present (Brill *et al.*, 2015; Svendsen *et al.*, 2016).

### Behavior and activity metrics

The behavior and activity of eight individuals (sharks from 2017) were measured while in the respirometer to understand how activity was affected by temperature and hypoxia. To quantify activity, individuals were fitted with the X16-4 mini accelerometer (Gulf Coast Data Concepts) attached to the first dorsal fin, which recorded triaxial acceleration at 25 Hz. The accelerometer weighed 27 g, which represented 0.35–1.1% of the body mass of the experimental sharks. Accelerometers were removed at the completion of each trial. We received a full acceleration data set for each 2017 trial except for three trials at 24°C where data were unreliable.

The static component (effect of gravity) in the x-, y- and z-axes were extracted from the raw acceleration values using a 3-s smoothing window, a time frame often used for the size of sharks in this study and a tailbeat cycle of approximately 1 Hz (Shepard *et al.*, 2008; Whitney *et al.*, 2016). The dynamic components in the x-, y- and z-axes were determined by subtracting the static component from the raw acceleration values. A wavelet analysis was then used to extract multiple activity metrics (Whitney *et al.*, 2016; Bouyoucos *et al.*, 2017): tailbeat frequency (TBF, number of tailbeats per second extracted from the dynamic component of the z-axis), tailbeat acceleration amplitude (TBAA, measure of amplitude of the acceleration wave also taken from the dynamic component of the z-axis) and overall dynamic body acceleration (ODBA, overall activity defined as sum of the dynamic components of all three axes). TBF, TBAA and ODBA were then averaged for each corresponding measurement period.

**Figure 2:**
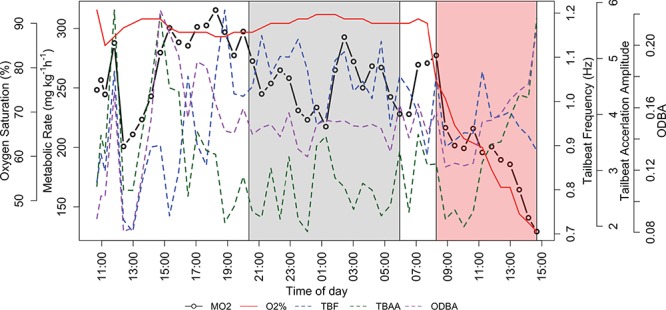
An example of a full trial from SB33 tested at 28°C. Each point corresponds to a calculated metabolic rate (MO_2_) along with the associated oxygen saturation (O_2_%), TBF, TBAA and ODBA values. The grey shaded area represents night hours created from sunrise/sunset time. The red shaded area represents the hypoxia period (O_2_% < 80%). S_crit_ for this individual at 28°C was 58% oxygen saturation.

### MMR and mRMR

Before the start of each trial, an individual was transferred out of the holding tank, fitted with an accelerometer (for some individuals, described above), placed in the respirometer and allowed 30 min to acclimate. Each experimental shark was exercised using our chase protocol, which consisted of 10 min of prodding to induce the animal to reach MMR (Lapointe *et al.*, 2014; Killen *et al.*, 2017). This time period was selected because after chasing the first shark of the experiments for 10 min, it stopped swimming. During another trial a shark stopped swimming after being chased for 6.5 min. Further, Marshall *et al.* (2015) found that in waters cooler than our experimental treatments (15–21°C) post-release mortality of sandbar sharks was 29%. Based on these accounts, to avoid mortalities (for a species that is considered to be overfished; SEDAR, 2017) and for ethical reasons, we erred on the side of caution and deemed 10 min to be a sufficient chase time. It is important to note this may have led to a slight underestimation of MMR of some individuals. Immediately following, the respirometer was sealed, and the first metabolic rate measurement was initiated. Metabolic rate measurements were then made for approximately 20 h to determine MMR and to allow individuals to recover (indicated by the metabolic rate leveling out) and reach mRMR.

### Critical oxygen saturation

Once mRMR had been established following the procedures described above, the hypoxia part of the trial was initiated, where metabolic rate measurements were taken as oxygen content was decreased in a stepwise fashion until the shark was no longer able to maintain its mRMR. S_crit_ was determined as the oxygen level at which the metabolic rate declined. After measuring at least three metabolic rates below S_crit_, the trial was terminated, and the oxygen was brought back to 90% saturation before the shark was transferred back into the holding tank. Figure 2 displays an example of a full trial.

### Data analysis

MMR was calculated by taking the mean of 10% of the highest metabolic rate measurements (3–4 measurements) during the entire normoxia period. This method was selected instead of using the common approach of taking the highest metabolic rate measurement because we wanted to ensure that MMR was not represented by an outlier measurement that may not have been the result of our chase protocol. In addition, unlike many non-obligate ram ventilators, we noticed that peak metabolic rate measurements often did not occur until further into the trial. This could be due to serological changes induced through the chase protocol that limit oxygen delivery mechanisms as further discussed below (Discussion). We calculated mRMR from the mean of 10% of the lowest metabolic rate measurements during the normoxic period to account for the varying number of measurements among individuals during that period of the trial (Norin *et al.*, 2014). AS was calculated by computing the difference between MMR and mRMR. To determine S_crit_, we found the first value where the shark’s metabolic rate dropped below mRMR and where the remaining metabolic rates were also below mRMR. Those metabolic rates were isolated, and a linear regression was applied to those values and the oxygen content associated with each value (ranged from 2 to 11 values). The oxygen content where the regression line and mRMR intersected was defined as C_crit_ (Schurmann and Steffensen, 1997). S_crit_ was calculated by dividing C_crit_ by the oxygen concentration at 100% saturation at the tested temperature. Lastly, C_crit_ was converted into critical oxygen partial pressure (P_crit_) by calculating the partial pressure of oxygen at 1 atmosphere for the start day of the trial in Wachapreague, VA, USA and multiplying by S_crit_.

**Figure 3:**
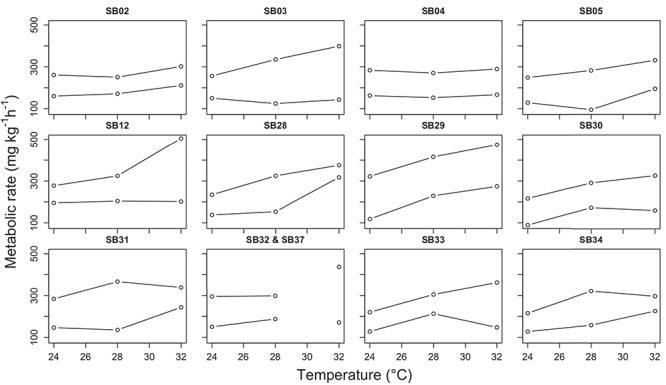
Measured MMR (top line in each plot) and mRMR (bottom line in each plot) for each shark tested in this study. The metabolic rate was measured at 24°C, 28°C and 32°C for each shark except for SB32 that was only measured at 24°C and 28°C (line connected those two data points). SB37 replaced SB32 at 32°C (single points).

A multivariate repeated-measures mixed effects model was developed in SAS 9.4 (SAS Institute) using the MIXED procedure to understand the effect of temperature on MMR, SMR, AS and P_crit_ (Lapointe *et al.*, 2014). The responses were MMR, SMR, AS and P_crit_ for each trial, the covariate was temperature and the random effect was individual fish. To maintain the assumption of normality, the four responses were multiplied by a constant so they would be on the same scale as MMR prior to being put in the model. We modeled the heterogeneity in responses among temperature treatments and specified the Kenward–Roger method for calculating the degrees of freedom (Kenward and Roger, 1997). Compound Symmetry, AR1 and Toeplitz correlation structures were fitted to the data, and the Bayesian Information Criterion (BIC) was used to identify the model with the most supported correlation structure (Littell *et al.*, 2006). Our model chosen for inference included a correlation structure of AR1. Lastly, a priori contrast statements of least-square means were generated using the LSMestimate statement in SAS to assess the effects of temperature on the four response variables. At the completion of the analysis, all model estimates were converted back to scale. All statistics were evaluated at significance levels of α = 0.05.

**Figure 4:**
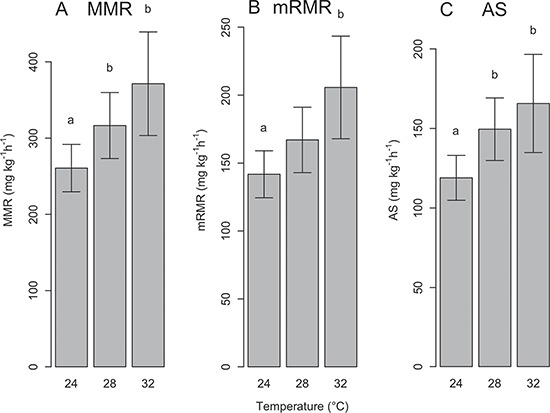
(**A**) MMR, (**B**) mRMR and (**C**) AS at 24°C, 28°C and 32°C of sandbar sharks when in normoxic waters. Data are model estimates of the mean ± 95% confidence interval (CI). Different lower case letters indicate a significant difference between two temperatures, whereas the lack of a letter indicates no significance.

To determine how temperature, hypoxia and shark activity together impacted metabolic rate, linear mixed effects models were fitted to data from sharks equipped with accelerometers. Separate models were applied to data obtained from the normoxic and hypoxic parts of the trials, with the former corresponding to the part of the trial when the oxygen saturation was 80% or higher and the latter when the oxygen saturation was below 80%. Potential covariates for the normoxic model included temperature, TBF, TBAA, ODBA, shark TL and the time since the start of trial. The full model was used to assess the need to model heterogeneity and correlation structure. Based on BIC, modeling the heterogeneity in responses among temperature treatments and using a correlation structure of AR1 were supported. A series of models was then developed using different combinations of the covariates mentioned above, and the model chosen for inference was selected using BIC. Potential covariates for the hypoxic model included temperature, oxygen content, TBF, TBAA, ODBA, TL as well as a calculated binary variable denoted as the crash metric. Since it was assumed that metabolic rate will differ before and after C_crit_ is reached, the crash metric consisted of a 1 for metabolic rate values during the hypoxic part of the trial that occurred prior to the shark reaching C_crit_ and a 0 for the metabolic rates that occurred after C_crit_ was reached. Similar to the normoxic model, the decision to model heterogeneity in responses among temperatures with an AR1 correlation structure was based on the full hypoxic model and ultimately supported through BIC. Multiple models were developed with various covariate combinations, and BIC was used for model selection. Predicted metabolic rates for the selected normoxic and hypoxic models were generated using estimated marginal means (Searle *et al.*, 1980). Estimates of uncertainty were generated from 1000 bootstrapped samples (Efron and Tibshirani, 1993). Predicted P_crit_ and associated uncertainty was converted back to C_crit_ and S_crit_. All linear mixed effects models were fitted using the nlme package in R v.3.4.3 (Pinheiro *et al.*, 2013).

**Table 2:** T statistics from the a priori contrast statements of least-square means generated from the multivariate repeated measures mixed effects model to assess the impact of temperature (24°C, 28°C and 32°C) on MMR, mRMR, AS and P_crit_

Metric	24°C × 28°C	24°C × 32°C	28°C × 32°C
MMR	*t*_(57.34)_ = −2.33^*^	*t*_(47.03)_ = −3.12^*^	*t*_(56.82)_ = −1.43
mRMR	*t*_(57.34)_ = −1.90	*t*_(47.03)_ = −3.25^*^	*t*_(56.11)_ = −1.81
AS	*t*_(57.34)_ = −2.81^*^	*t*_(47.03)_ = −2.90^*^	*t*_(56.11)_ = −0.93
P_crit_	*t*_(56.82)_ = 0.14	*t*_(46.93)_ = −1.29	*t*_(59.02)_ = −1.25

The degrees of freedom are in parentheses. The (^*^) signifies a significant difference between the two temperatures.

## Results

### MMR, mRMR, AS and critical oxygen partial pressure

**Figure 5:**
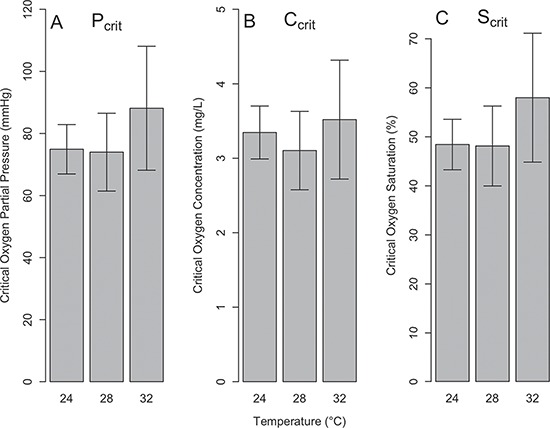
(**A**) Critical oxygen partial pressure (P_crit_), (**B**) critical oxygen concentration (C_crit_) and (**C**) critical oxygen saturation (S_crit_) of sandbar sharks at 24°C, 28°C and 32°C. P_crit_ data are model estimates of the mean ± 95% CI. C_crit_ and S_crit_ were calculated from P_crit_ model estimates. The lack of a letter indicates no significance.

The multivariate repeated measures mixed effects model was successfully fitted, and evaluation of diagnostics (e.g. plots of residuals for each response variable) showed reasonable goodness-of-fit. Although there was high variability in measured MMR, mRMR and AS among individuals (Fig. 3), model results indicated that differences were evident for these three metrics among temperatures. Statistically significant differences were detected over the three experimental temperatures, primarily between 24°C and 28°C and between 24°C and 32°C (Fig. 4, Table 2). Specifically, MMR increased by 21% and 42% from 24°C to 28°C and 32°C, respectively (Fig. 4A; Table 2), while mRMR increased by 18% from 24°C to 28°C and 45% from 24°C to 32°C (Fig. 4B; Table 2). Similar to MMR, AS increased considerably from 24°C to 28°C (26%) and 24°C to 32°C (39%; Fig. 4C; Table 2). In contrast to the other metrics analyzed, mean P_crit_ only increased by 18% and 19% when comparing 24°C and 28°C to 32°C, respectively, and no significant differences were detected (Fig. 5A; Table 2). Plots of the raw data for MMR, mRMR, AS, S_crit_ and C_crit_, are presented in the Supplementary Data.

**Figure 6:**
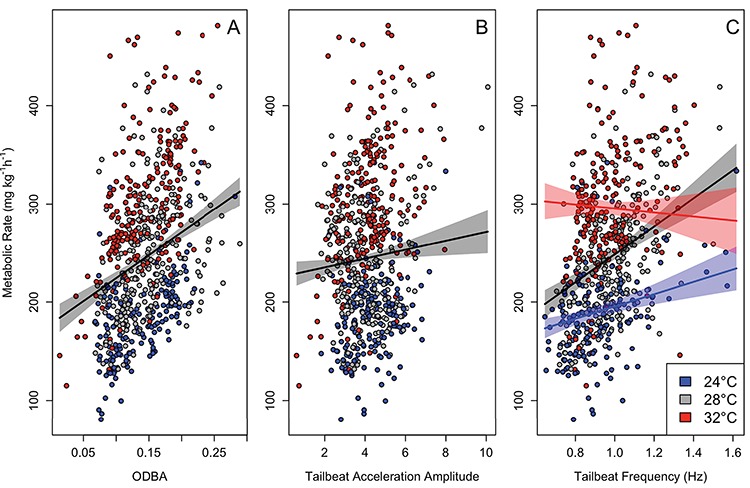
Metabolic rate values by ODBA (**A**), TBAA (**B**) and TBF (**C**) when sandbar sharks are exposed to normoxic waters. The colored circles represent the observed metabolic rate values for a given temperature. The line and associated shaded region represent the estimated metabolic rate values and the 95% CI, respectively. An interaction did not occur between temperature and ODBA (A) or TBAA (B); therefore, temperature was combined when estimating metabolic rate for those metrics. However, because there was a significant interaction between temperature and TBF, there are separate estimated metabolic rates for each temperature that are represented by different colored lines and shaded regions in C.

### Normoxia

The normoxic model with the most empirical support included ODBA, TBAA, time since chase and an interaction between TBF and temperature. We concluded that each of these covariates was important in explaining variation in the metabolic rate data (see ∆BIC table in Supplementary Table 1). Predicted metabolic rate increased by 47 ± 6 mg O_2_ kg^−1^h^−1^ for every 0.1 unit increase in ODBA (Fig. 6A), whereas metabolic rate only increased by 5 mg O_2_ kg^−1^h^−1^ for every unit increase in TBAA (Fig. 6B). For every hour increase since the animal was chased, the metabolic rate dropped approximately 3 mg O_2_ kg^−1^h^−1^. The estimated effect in metabolic rate over TBF differed among the levels of temperature considered. That is, for every unit increase in TBF, metabolic rate increased 63 and 142 mg O_2_ kg^−1^h^−1^ for 24°C and 28°C, respectively. However, at 32°C, metabolic rate actually decreased by 20 mg O_2_ kg^−1^h^−1^ for every unit increase in TBF (Fig. 6C). Lastly, it is important to note that during three trials at 32°C during normoxia, we documented periods of time when sharks intermittently stopped swimming, ranging from a few seconds to over an hour.

### Hypoxia

**Figure 7:**
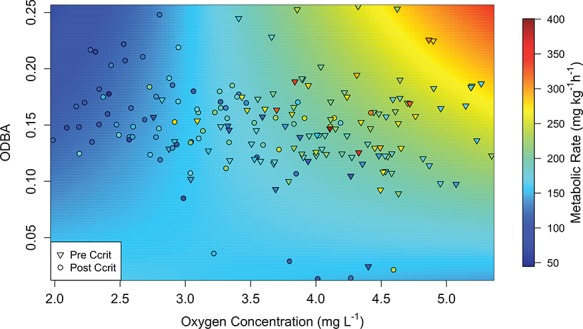
Metabolic rate in relation to ODBA and oxygen concentration during hypoxia (<80% oxygen saturation). Symbols represent observed data, where ∇ are before C_crit_ was reached and Ο are after C_crit_ was reached. The colors on the symbols represent the observed metabolic rate. The surface behind the symbols represents the predicted metabolic rate estimated from the model.

The model that provided the most parsimonious description of sandbar shark metabolic rate under hypoxic conditions included TBF, temperature and an interaction between ODBA and oxygen content (see ∆BIC table in Supplementary Table 2). Predicted metabolic rate increased by 55 ± 20 mg O_2_ kg^−1^h^−1^ for every unit increase in TBF. In hypoxic water, metabolic rate differed among temperatures such that as temperature increased from 24°C to 28°C to 32°C, metabolic rate increased from 149 ± 9 to 183 ± 6 to 217 ± 13 mg O_2_ kg^−1^h^−1^, respectively. Lastly, as ODBA and oxygen content increased, particularly at higher oxygen concentrations (>3.5 mg l^−1^), predicted metabolic rate also increased. However, as oxygen decreased, particularly at lower oxygen concentrations (<3.5 mg l^−1^) and ODBA increased, predicted metabolic rate decreased (Fig. 7). Often during hypoxic conditions (<80% oxygen saturation), sharks displayed a banking behavior where they would swim along the edge of the respirometer with its ventral side facing the side. We also observed during hypoxia, two trials at 24°C, three trials at 28°C and four trials at 32°C periods of time when sharks either stopped swimming completely (in this case the trial was terminated) or intermittently stopped swimming.

## Discussion

To the best of our knowledge, this is the first study to measure AS and P_crit_ in an obligate ram-ventilating elasmobranch. In addition, the size of our respirometer allowed us to measure the metabolic rate and activity of juvenile sandbar sharks across a wide range of sizes (79.5–113.5 cm TL). We were also able to use the various activity measurements to better understand environmental thresholds of sandbar sharks. Our findings lead us to suggest that caution should be exercised when using activity to estimate field metabolic rate because, as demonstrated in this study, the correlative relationship between activity and metabolic rate breaks down at high temperatures and low oxygen levels.

### MMR, mRMR, AS and normoxia

Referenced model response variables (e.g. mRMR, AS, metabolic rate, etc.) below represent model predictions, while raw values are indicated as such. As expected, mRMR values in the present study were higher than the SMR measurements reported by Dowd *et al.* (2006) for immobile individuals (91 ± 4 mg O_2_ kg^−1^h^−1^ at 24°C; 125 ± 7 mg O_2_ kg^−1^h^−1^ at 28°C). When compared to other obligate ram ventilators, such as the tuna species (Korsmeyer and Dewar, 2001), sandbar shark AS was substantially lower. Contributing to this trend may be the significantly smaller gill surface area observed in sandbar sharks compared to other obligate ram-ventilating teleosts and elasmobranchs (Emery and Szczepanski, 1986). In addition, with a diet consisting of mostly benthic crustaceans and fishes (Ellis and Musick, 2007), juvenile sandbar sharks may not require a high AS.

The variation in raw AS among individual juvenile sandbar sharks over the experimental temperature regime suggests that the potential underlying mechanisms describing AS may differ from individual to individual. For example, some individuals displayed a bell-shaped curve in AS over temperature (e.g. SB28, SB31 and SB34), some showed an increase in AS with temperature (e.g. SB03, SB12 and SB33) and others displayed very little difference in AS over temperature (e.g. SB02, SB04 and SB29) (Fig. 3). Due to this high variability, particularly at 32°C, it is difficult to broadly determine if our data support the OCLTT theory (i.e. maximum AS occurs at the optimal temperature) or if sandbar shark AS increases until lethal temperature limits. In individuals that displayed a bell-shaped curve, maximum AS and thus optimal performance occurred at 28°C (24°C < T_optAS_ < 32°C). Whereas for those individuals that maximized AS at 32°C, we assume AS would decline above 32°C followed by mortality shortly thereafter. This would suggest that performance or fitness is not optimized at maximum AS for those individual sharks. Clark *et al.* (2013) suggested that some species display multiple performances–multiple optima, where there are separate optimal temperatures for different physiological processes (e.g. growth, reproduction and digestion). However, it is difficult to determine if our results support this theory because the optimal temperatures for those processes are unknown for sandbar sharks. The variability in the data may be the result of differences in activity, physical fitness, adaptation and physiological processes among individuals. Variation in AS may have also resulted from a potential slight underestimation of MMR in some individuals in an effort to avoid mortalities during or following the chase protocol. Further, because temperature could not be controlled in the holding tank and all individuals were tested in the same order of increasing temperature treatments, the relationships of the measured metrics could potentially be affected. For example, sharks may be more acclimated to the chase protocol or the respirometer for subsequent experiments, which could impact MMR and mRMR (Clark *et al.*, 2013). Lastly, variation in acclimation time for individuals in particular when exposed to 32°C could have affected metabolic rate metrics. Although we were unable to control these aforementioned conditions, it is possible that they contributed to the individual variation in these metrics observed here.

Clearer trends were evident when both metabolic rate and activity were considered. Under normoxic conditions, the expected correlation between metabolic rate and activity (Bouyoucos *et al.*, 2017; Lear *et al.*, 2017) broke down at 32°C, where the metabolic rate of sandbar sharks actually slightly declined as activity increased (Fig. 6). At high temperatures, sandbar sharks have to delicately balance the increase in oxygen received from increasing their TBF to improve ram ventilation against the energy expenditure needed for locomotion. This is evident when some sharks actually intermittently stopped swimming for periods of time under normoxic conditions at 32°C. A decline in shark performance at 32°C may be due to the physiological stress incurred from exercise, acute stress, increased demand for oxygen and/or decreased oxygen in the water. These stressors are known to cause acidosis and hyperkalemia in fishes (Cliff and Thurman, 1984; Brill *et al.*, 2008; Skomal and Mandelman, 2012). It is well known that acidosis and hyperkalemia impact muscle performance, impairing cardiovascular function and potentially reducing stroke volume at a time when a shark may actually need to increase stroke volume to increase oxygen delivery (Farrell *et al.*, 2009; Norin *et al.*, 2014).

### Critical oxygen partial pressure and hypoxia

Based on the P_crit_ values, juvenile sandbar sharks are not hypoxia tolerant. Regardless of temperature, P_crit_ was above those reported for other teleost and elasmobranch species. For example, striped bass (*Morone saxatilis*), a common fish species in Chesapeake Bay, has an S_crit_ of 35 ± 2% and C_crit_ of 2.5 ± 0.2 mg l^−1^, at 28°C (Lapointe *et al.*, 2014), both substantially lower than the S_crit_ (51 ± 2%) and C_crit_ (3.3 ± 0.2 mg l^−1^) of juvenile sandbar sharks at the same temperature (Fig. 4B and C). When compared to elasmobranch fishes where P_crit_ was measured, such as the epaulette shark (*Hemiscyllium ocellatum*, 38 ± 3 mmHg) and shovelnose ray (*Aptychotrema rostrate*, 54 ± 3 mmHg), the juvenile sandbar shark’s P_crit_ values are likewise substantially higher (Speers-Roesch *et al.*, 2012). When compared to another obligate ram-ventilating species, such as the yellowfin tuna at 25°C (hypoxia tolerance of 3.7 mg l^−1^; Bushnell and Brill, 1991; Bernal *et al.*, 2017), sandbar sharks have a similar hypoxia tolerance. The high P_crit_ values in juvenile sandbar sharks are likely the result of this species also being (like many carcharhinid species) an obligate ram ventilator (Carlson *et al.*, 2004; Dowd *et al.*, 2006). In hypoxia, obligate ram ventilator species rely on increasing their swimming speed or mouth gape to counteract a decline in oxygen in the water (Dizon, 1977; Bushnell and Brill, 1991; Carlson and Parsons, 2001); however, once P_crit_ is reached, obligate ram-ventilating fishes will not be able to maintain minimum swimming speeds to ventilate their gills adequately or to maintain hydrostatic equilibrium (Carlson and Parsons, 2001). Although, P_crit_ did not differ substantially among temperatures, the mean P_crit_ at 32°C was the highest when compared to means at 24°C and 28°C, which was expected because as temperature increases, the demand for oxygen increases and the solubility of oxygen in seawater decreases (Schurmann and Steffensen, 1997). The lack of a substantial difference was the result of high amount variability in P_crit_ values, particularly at 32°C (Fig. 5A). Sandbar shark metabolic rate did increase as temperature increased in hypoxic waters (<80% oxygen saturation), suggesting that at warmer temperatures (~32°C), the demand for oxygen is higher while in less oxygenated water, which can lead to increased stress and a higher risk of mortality. An alternative hypothesis is that juvenile sandbar sharks are adapted to large tidal fluctuations in temperature (4.6°C; Kelley, unpublished data); therefore, the ability to deliver oxygen to the tissues is not compromised due to increased temperatures.

In hypoxic waters, sandbar shark metabolic rate was highly dependent on oxygen level and the shark’s activity. The synergistic effect of oxygen and activity on metabolic rate led to different trends above and below ~3.5 mg l^−1^, which was similar to the mean (± standard error (SE)) C_crit_ of sandbar sharks between 24°C and 32°C (3.4 ± 0.1 mg l^−1^). The positive correlation of metabolic rate and activity, as oxygen concentrations increased from 3.5 mg l^−1^, followed trends similar to those when sharks were under normoxic conditions (>80% oxygen saturation). When oxygen dropped below 3.5 mg l^−1^, however, an inverse relationship occurred between metabolic rate and activity, a trend that was also evident at 32°C under normoxic conditions. The breakdown in the positive relationship between metabolic rate and activity suggests that at these lower oxygen levels, sandbar sharks employ anaerobic metabolism to power swimming, which can lead to acidosis and hyperkalemia. It is clear this behavior cannot be sustained, based on the many periods of time when sharks stopped swimming either completely or intermittently during hypoxia. According to our data the oxygen concentration threshold for juvenile sandbar sharks is ~3.5 mg l^−1^, and this threshold is positively correlated with temperature.

### Climate change impacts

It is critical to understand environmental thresholds for marine species amid climate change. Although sandbar sharks are not expected to encounter 32°C waters often, the potential to encounter these temperatures will increase as climate change continues to alter marine environments, particularly within coastal habitats. With an increase in temperature we also expect to see an increase in the extent and severity of hypoxic waters that may have a large impact on the obligate ram-ventilating sandbar shark. For example, along the eastern shore of Virginia, where known sandbar shark nursery habitat exists, water temperatures during the months of July and August during 2016 and 2017 exceeded 32°C at times (range: 21.3–32.8°C; mean: 27.4°C; Kelley, unpublished data) and oxygen levels ranged from 2.9 mg l^−1^ to 8.2 mg l^−1^. By the mid-21st century, Chesapeake Bay, which is the largest sandbar shark nursery habitat in the USA, is expected to increase 1.75°C relative to the mid-1990s (Muhling *et al.*, 2018). In addition, by the end of the century, heat waves are also projected to increase by over two standard deviations along the Mid-Atlantic (Najjar *et al.*, 2010). Also by 2050, Chesapeake Bay is predicted to have the largest increase in cumulative hypoxic volume (72–202 km^3^ days) at oxygen concentrations between 2–5 mg l^−1^ (Irby *et al.*, 2018), a range of oxygen concentrations that include the P_crit_ of juvenile sandbar sharks. This volume of hypoxic water is expected to occur earlier in the summer as well (Irby *et al.*, 2018). These environmental changes will likely significantly diminish the suitability of Chesapeake Bay and adjacent coastal areas to serve as nursery habitat for sandbar sharks in the western Atlantic. As climate change impacts worsen, juvenile sandbar sharks, which can spend up to 10 years in these nursery habitats, may see available habitat reduced and be forced to seek out novel nursery habitats or risk increases in juvenile mortality. This may, in turn, affect the overall abundance of an already overfished sandbar shark population (SEDAR, 2017). At an ecological level, juvenile sandbar sharks are top predators within coastal habitats and may control the populations of other fish species (Ellis and Musick, 2007). Shifts in juvenile sandbar shark distribution could, therefore, also have negative effects on the population of lower trophic species within these habitats.

## Conclusions

This study was able to identify the temperature and oxygen thresholds of juvenile sandbar sharks. We found that their performance substantially declines at 32°C (even in normoxia) and at oxygen concentrations below 3.5 mg l^−1^ and that activity becomes inversely correlated with metabolic rate under high-stress conditions. These impacts suggest that in the face of climate change, areas of Chesapeake Bay may become less suitable nursery habitat for sandbar sharks. It is critical for future studies to use environmental thresholds like those identified in this study to predict species distribution under climate change scenarios to understand potential habitat shifts.

## Supplementary Material

conphys-2018-118_coz026
