# Supplementary material for: The impacts of warming and hypoxia on the performance of an obligate ram ventilator
Source: Conserv Physiol. 2019 Jun 19;7(1):coz026. doi: 10.1093/conphys/coz026 (PMC6656321; doi:10.1093/conphys/coz026)
Supplement: conphys-2018-118_coz026 [file conphys-2018-118_coz026.pdf]

OXFORD UNIVERSITY PRESS | Conservation Physiology

The impacts of warming and hypoxia on the performance of an obligate ram ventilator

Journal:	*Conservation Physiology*
Manuscript ID	CONPHYS-2018-118.R2
Manuscript Type:	Research Article
Date Submitted by the Author:	23-Apr-2019
Complete List of Authors:	Crear, Daniel; Virginia Institute of Marine Science, Fisheries Science<br>Brill, Richard; Virginia Institute of Marine Science, Fisheries Science<br>Bushnell, Peter; Indiana University South Bend, Department of Biological Sciences<br>Latour, Robert; Virginia Institute of Marine Science, Fisheries Science<br>Schwieterman, Gail; Virginia Institute of Marine Science, Fisheries Science<br>Steffen, Rachel; Washington and Lee University, Biology<br>Weng, Kevin Chi-Ming; Virginia Institute of Marine Science, Fisheries Science
Keywords:	respirometry, accelerometer, aerobic scope, critical oxygen concentration, climate change, sandbar shark

SCHOLARONE™
Manuscripts

The impacts of warming and hypoxia on the performance of an obligate ram ventilator

Daniel P. Crear, Fisheries Science Department, Virginia Institute of Marine Science, College of William & Mary, Gloucester Point, VA 23062, USA
Rich W. Brill, Fisheries Science Department, Virginia Institute of Marine Science, College of William & Mary, Gloucester Point, VA 23062, USA
Peter G. Bushnell, Department of Biological Sciences, Indiana University South Bend, South Bend, IN 46634, USA
Robert J. Latour, Fisheries Science Department, Virginia Institute of Marine Science, College of William & Mary, Gloucester Point, VA 23062, USA
Gail D. Schwieterman, Fisheries Science Department, Virginia Institute of Marine Science, College of William & Mary, Gloucester Point, VA 23062, USA
Rachel M. Steffen, Biology Department, Washington & Lee University, Lexington, VA 244150
Kevin C. Weng, Fisheries Science Department, Virginia Institute of Marine Science, College of William & Mary, Gloucester Point, VA 23062, USA

Corresponding author: Daniel Crear
Phone Number: (804) 684-7819
Email: dcrear8@gmail.com

For Review Only

Lay Summary
We assessed the impact of warming and hypoxia on the metabolic rate and behavior of an obligate ram-ventilating marine predator, the sandbar shark, that uses coastal habitat as a nursery ground. Based on their thermal threshold and low hypoxia tolerance, we expect sandbar sharks to be negatively impacted by climate change.

Total Word Count: 8934 (includes all sections)

Abstract

Climate change is causing the warming and deoxygenation of coastal habitats like Chesapeake Bay that serve as important nursery habitats for many marine fish species. As conditions continue to change, it is important to understand how these changes impact individual species' behavioral and metabolic performance. The sandbar shark (*Carcharhinus plumbeus*) is an obligate ram ventilating apex predator whose juveniles use Chesapeake Bay as a nursery ground ~~for~~ up to 10 years of age. The objective of this study was to measure juvenile sandbar shark metabolic and behavioral performance as a proxy for overall performance (i.e., fitness or success) when exposed to warm and hypoxic water. Juvenile sandbar sharks (79.5 – 113.5 cm TL) were collected from an estuary along the eastern shore of Virginia and returned to lab where they were fitted with an accelerometer, placed in a respirometer, and exposed to varying temperatures and oxygen levels. Juvenile sandbar shark overall performance declined substantially at 32°C or when dissolved oxygen concentration was reduced below 3.5 mg l^{-1} (51% oxygen saturation between 24 – 32°C). As the extent of warm hypoxic water increases in Chesapeake Bay, we expect that the available sandbar shark nursery habitat will be reduced, which may negatively impact the population of sandbar sharks in the western Atlantic~~,~~ as well as the overall health of the ecosystem within Chesapeake Bay.

For Review Only

Introduction

Climate change is warming coastal areas and estuaries worldwide. An increase in anthropogenic nutrient input is likewise increasing the severity and extent of hypoxic episodes in many of these areas (Conley *et al.*, 2007, Hagy *et al.*, 2004, Irby *et al.*, 2016, Kemp *et al.*, 2005, Najjar *et al.*, 2010, Preston, 2004, Rabalais *et al.*, 2009). These conditions are expected to worsen over the next 100 years as climate change impacts are exacerbated (Irby *et al.*, 2018, Najjar, et al., 2010, Rabalais, et al., 2009).

Changing environmental conditions in coastal areas and estuaries are likely to impact marine fish species that rely on these habitats as primary nursery grounds (Rijnsdorp *et al.*, 2009). For example, while in their nursery habitat, juvenile weakfish (*Cynoscion regalis*) avoided waters in a tributary of Indian River Bay, DE, USA with dissolved oxygen below 2 $mg\ l^{-1}$ (Tyler and Targett, 2007). The prevalence of juvenile bull sharks (*Carcharhinus leucas*) has actually increased in their nursery ground habitat within Pamlico Sound, NC, USA over the last decade as a result of increased water temperatures (Bangley *et al.*, 2018). Shifts in species distribution, similar to the examples above, can lead to changes in the timing of migration of juveniles and adults (Nye *et al.*, 2009, Turner *et al.*, 2015), reproductive patterns of adults (Last *et al.*, 2011), and abundance of all life stages (Last, et al., 2011, Lynch *et al.*, 2014); all of which can influence population level success and the overall health of ecosystems (Morley *et al.*, 2018).

To understand these potential changes in fish ecology, it is important to assess the relationship between environmental conditions and the performance of individual fish. Performance is often assessed through measurement of aerobic scope (AS, i.e., the difference

For Review Only

between maximum and standard metabolic rates; Claireaux & Lefrançois 2007, Di Santo 2016) , which quantifies an individual's metabolic power (i.e., energy use per unit time) under which all of life's processes beyond basic maintenance (e.g., growth, reproduction, digestion, movement) must occur (Clark *et al.*, 2013). Consistent with the theory of oxygen- and capacity-limited thermal tolerance (OCLTT), AS is a measure of fitness and performance in ectotherms. According to OCLTT, AS follows a bell-shaped curve with temperature such that there is a temperature where AS is optimized (Clark, et al., 2013, Fry, 1971). Therefore, it is expected that long term warming will reduce AS and decrease the ability for many individuals to carry out multiple life processes simultaneously unless individuals adjust their range and distribution (Pörtner and Knust, 2007). However, the theory of OCLTT does not hold true for all species, as some demonstrate an increase in aerobic scope with temperature until temperature approaches lethal levels (Clark, et al., 2013, McKenzie *et al.*, 2016). This suggests that, for some species, the temperature at which AS is optimized is not equivalent to the temperature at which performance is maximized (Lefevre, 2016).

Increases in the extent and severity of hypoxic episodes in coastal areas are affecting the physiology and thus ecology of coastal species (Diaz and Rosenberg, 1995, Ludsin *et al.*, 2009, Tyler and Targett, 2007, Wannamaker and Rice, 2000, Zhang *et al.*, 2009). Hypoxia tolerance is often quantified by measurement of critical oxygen levels or oxygen level at which individuals can no longer maintain standard metabolic rate (SMR). The critical oxygen saturation (S_{crit}) should increase with temperature due to increases in SMR and decreases in oxygen solubility in water. Individuals cannot occupy waters long term with an oxygen level below S_{crit} because, under these conditions, at least some of the power needed to maintain homeostasis must be met through anaerobic metabolism (Brill *et al.*, 2015, Fry and Hart, 1948, Schurmann and Steffensen,

For Review Only

1997). Similar to S_{crit}, the quantities C_{crit} and P_{crit} are the concentration and partial pressure of oxygen, respectively, at which a fish can no longer maintain SMR. All of these measures of critical oxygen, as well as AS, can be determined through intermittent-flow respirometry (Clark *et al.*, 2011, Lapointe *et al.*, 2014).

Measuring metabolic performance (i.e., AS) of an obligate ram ventilator, like a tuna or some shark species, is difficult because of the necessity of individuals to swim constantly and, therefore, a need to measure activity simultaneously. This requires either a swim tunnel respirometer where swimming speed can be controlled, or a respirometer large enough for fish to swim independently coupled with a method to quantify activity. Since many species exhibit difficulty swimming in a swim tunnel, a large circular tank can be used as the respirometer such that fish are able to swim freely (Lear *et al.*, 2017). Because the swimming behavior and activity of fish cannot be controlled in the large circular respirometer~~,~~ accelerometers have been used to measure activity during experimentation (Lear, et al., 2017). Activity measurements (i.e., behavioral performance), obtained through ~~measured from~~ accelerometers (Whitney *et al.*, 2007, Whitney *et al.*, 2016), ~~,~~ can be used as a performance metric to quantify mechanical work, describe behavior (Gleiss *et al.*, 2011, Payne *et al.*, 2018), and be used as an indicator to understand locomotor performance under different environmental regimes (Payne, et al., 2018, Payne *et al.*, 2016). Activity is correlated with metabolic performance, which suggests that accelerometers can be used to infer field metabolic rate when applied to free-ranging individuals (Bouyoucos *et al.*, 2017, Lear, et al., 2017). However, the relationship between accelerometer derived activity metrics and metabolic rate has not been assessed under high levels of environmental stress.

For Review Only

The sandbar shark (*Carcharhinus plumbeus*) is an obligate ram ventilating species that relies on coastal habitats as nursery grounds during younger life stages. In late spring, pupping occurs in Chesapeake Bay and coastal estuaries along the mid-Atlantic, where young-of-year (YOY) remain through summer (Conrath and Musick, 2007, Grubbs and Musick, 2007). After moving south or offshore during winter, juveniles return to these nursery areas to forage and avoid larger predators during summer for the following 4-10 years (Grubbs and Musick, 2007). As a result of climate change and anthropogenic nutrient input, Chesapeake Bay is becoming warmer and more hypoxic (Hagy, et al., 2004, Kemp, et al., 2005, Najjar, et al., 2010, Preston, 2004). Temperature and oxygen limitations are not well understood for juvenile sandbar sharks. Therefore, the objective of this study was to measure juvenile sandbar shark metabolic and behavioral performance as a proxy of overall performance (i.e., fitness or success) when exposed to warm and hypoxic water.

For Review Only

Methods

Shark Collection and Maintenance

Thirteen juvenile sandbar sharks (79.5 – 113.5 cm TL; 2.6 – 7.8 kg) were collected along the eastern shore of Virginia and brought back to the Virginia Institute of Marine Science (VIMS) Eastern Shore Lab (ESL), Wachapreague, VA, USA during the summers of 2016 and 2017 (Table 1). Individuals were held in a fiberglass tank (6.1 m diameter, 45,000 l) with a flow-through seawater system and acclimated to captivity for at least two weeks prior to experimentation. Sharks were fed frozen menhaden (*Brevoortia tyrannus*) until satiation every 3-5 days. All protocols for shark sampling, handling, and experimentation were approved by the

College of William and Mary Institutional Animal Care and Use Committee (protocol no. IACUC-2017-05-26-12133-kcweng).

Experimental Design

Maximum metabolic rate (MMR), minimum routine metabolic rate (mRMR; a proxy for SMR because sandbar sharks are obligate ram ventilators), AS, and S_{crit} were measured in 12 sharks at 24, 28, and 32°C using intermittent-flow respirometry to understand the effects of warming and hypoxia on sandbar shark physiology (Brill, et al., 2015, Lapointe, et al., 2014). Eleven sharks were tested at all three treatments (24, 28, and 32°C), while data collected at 24 and 28°C from one shark was combined with data collected at 32°C from a different shark to represent one set of temperature treatments due to a mortality suffered in the holding tank between experiments. Sharks were acclimated to treatment temperatures in their holding tank through natural increases in water temperature throughout the summer (range: 18.0 - 33.8°C) because we were unable to control temperature in our holding tank. However, by using this approach we were able to maximize ecological relevance. When experimental temperatures exceeded holding tank temperatures, individuals were transferred to a separate tank at the experimental temperature for at least 24 - 48 h prior to being introduced into the respirometer. The respirometry system consisted of two fiberglass tanks (2.4 m diameter, 1500 l volume) such that the first served as the respirometer and the second was a water reservoir used to flush the respirometer. To reduce the volume of the respirometer and ensure each experimental shark would swim in a circle, a negatively buoyant, smaller tank was placed in the center of the respirometer to create a circular track. A clear plastic sheet was placed over the respirometer and ~~synched~~ cinched to the side of the tank to prevent air-water gas exchange. Temperature was

For Review Only

controlled by a large chiller unit with the addition of a heater kit (Teco TK 6000 Aquarium Chiller, Aquatic Solutions), while oxygen levels were controlled by bubbling air (normoxia) or nitrogen gas (hypoxia) into the water. The two tanks were connected through a drain on the bottom and PVC tubing over the top (Fig. 1A, B). Oxygen levels in the respirometer and reservoir were monitored every second using a temperature compensated, two-channel FireSting oxygen meter (Pyroscience) equipped with fiber optic oxygen probes that were fixed along the side of the respirometer and reservoir. Output from the oxygen probe in the respirometer was recorded in the FireStingO_2 software and relayed to a program designed in Dasylab 9.02 (National Instruments) specifically for repeatedly recording metabolic rate measurements in intermittent flow respirometry (Brill, et al., 2015, Lapointe, et al., 2014). The program also controlled the flow of nitrogen through a solenoid valve to maintain precise oxygen levels using output from the oxygen probe in the reservoir.

During a trial, the respirometry system cycled between the flushing and measurement period. During the flushing period (ranged from 30 – 45 min), oxygen and temperature controlled water was pumped up and out of the reservoir, into the respirometer, and through a diffuser at the bottom of the respirometer (Fig. 1B) to ensure thorough mixing. Water flowed back into the reservoir through the bottom drain between the two tanks, allowing a continuous exchange of water between the two tanks during the flushing period. During the 15 min measurement period, the flush pump was turned off and the shark's oxygen consumption reduced the oxygen in the respirometer. We assumed the shark's swimming motion adequately mixed the water in the respirometer during trials. The measurement period consisted of a three min equilibration interval (to ensure oxygen mixing in the respirometer) followed by 12 min of data

For Review Only

recording to measure the rate of oxygen decline. The slope of a linear regression model fitted to the oxygen measurements was used to calculate metabolic rate using the equation:

$$MO_2 = b\ x\ V\ x\ W^{-1}$$

where MO_2 = metabolic rate (mg O_2 kg^{-1} h^{-1}), b = rate of change of oxygen content (estimated slope of linear regression) over the 12 min recording period (s^{-1}), V = respirometer volume (l) corrected for the volume of the shark, and W = weight of the fish (kg). Metabolic rate measurements where the regression R^2 value was below 0.8 were eliminated and assumed to be compromised due to either poorly mixed water in the respirometer or contact between the shark and oxygen probe (which did occur occasionally).

To account for microbial respiration, oxygen consumption measurements were made in the absence of an experimental shark for at least 3 h prior to, and after, each trial. Based on those oxygen consumptions, a linear regression was used to estimate the rate of oxygen decline due to microbial respiration during the trial. The estimated oxygen consumptions were then subtracted from the measured rates of oxygen decline when the shark was present (Brill, et al., 2015, Svendsen *et al.*, 2016).

Behavior and Activity Metrics

The behavior and activity of ~~a~~ eight individuals (sharks from 2017) were measured while in the respirometer to understand how activity was affected by temperature and hypoxia. To quantify activity, individuals were fitted with the X16-4 mini accelerometer (Gulf Coast Data Concepts) attached to the first dorsal fin, which recorded triaxial acceleration at 25 Hz. The accelerometer weighed 27 g, which represented 0.35-1.1% of the body mass of the experimental sharks. Accelerometers were removed at the completion of each trial. We received a full

For Review Only

acceleration data set for each 2017 trial except for three trials at 24°C where data were unreliable.

The static component (effect of gravity) in the x, y, and z-axes were extracted from the raw acceleration values using a 3 s smoothing window, a time frame often used for the size of sharks in this study and a tailbeat cycle of approximately 1 Hz (Shepard *et al.*, 2008, Whitney, et al., 2016). The dynamic components in the x, y, and z-axes were determined by subtracting the static component from the raw acceleration values. A wavelet analysis was then used to extract multiple activity metrics (Bouyoucos, et al., 2017, Whitney, et al., 2016): tailbeat frequency (TBF, number of tailbeats per second extracted from the dynamic component of the z-axis), tailbeat acceleration amplitude (TBAA, measure of amplitude of the acceleration wave also taken from the dynamic component of the z-axis), and overall dynamic body acceleration (ODBA, overall activity defined as sum of the dynamic components of all three axes). TBF, TBAA, and ODBA were then averaged for each corresponding measurement period.

Maximum and Minimum Routine Metabolic Rate

Before the start of each trial, an individual was transferred out of the holding tank, fitted with an accelerometer (for some individuals, described above), placed in the respirometer, and allowed 30 mins to acclimate. Each experimental shark was exercised using our chase protocol, which consisted of 10 min of prodding to induce the animal to reach MMR (Killen *et al.*, 2017, Lapointe, et al., 2014). This time period was selected because after chasing the first shark of the experiments for 10 min, it stopped swimming. During another trial a shark stopped swimming after being chased for 6.5 mins. Further, Marshall et al. 2015 found that in waters cooler than our experimental treatments (15-21°C) post release mortality of sandbar sharks was 29%. Based on

For Review Only

these accounts, to avoid mortalities (for a species that is considered to be overfished; SEDAR 2017) and for ethical reasons, we aired erred on the side of caution and deemed 10 mins to be a sufficient chase time. It is important to note this may have led to a slight underestimation of MMR of some individuals. Immediately following, the respirometer was sealed and the first metabolic rate measurement was initiated. Metabolic rate measurements were then made for approximately 20 h to determine MMR and to allow individuals to recover (indicated by the metabolic rate leveling out) and reach mRMR.

Critical Oxygen Saturation

Once mRMR had been established following the procedures described above, the hypoxia part of the trial was initiated, where metabolic rate measurements were taken as oxygen content was decreased in a stepwise fashion until the shark was no longer able to maintain its mRMR. S_{crit} was determined as the oxygen level at which the metabolic rate declined.a decline in metabolic rate was evident. After measuring at least three metabolic rates below S_{crit}, the trial was terminated, and the oxygen was brought back to 90% saturation before the shark was transferred back into the holding tank. Fig. 2 displays an example of a full trial.

Data Analysis

MMR was calculated by taking the mean of 10% of the highest metabolic rate measurements (3-4 measurements) during the entire normoxia period. This method was selected instead of using the common approach of taking the highest metabolic rate measurement because we wanted to ensure that MMR was not represented by an outlier measurement that may not have been the result of our chase protocol. In addition, unlike many non-obligate ram ventilators,

For Review Only

we noticed that peak metabolic rate measurements often did not occur until further into the trial. This could be due to serological changes induced through the chase protocol~~,~~ which limit oxygen delivery mechanisms as further discussed below (Discussion). ~~as a result of the chase protocol, may have been due to the shark's oxygen delivery being compromised.~~ We calculated mRMR from the mean of 10% of the lowest metabolic rate measurements during the normoxic period to account for the varying number~~amount~~ of measurements among individuals during that period of the trial (Norin *et al.*, 2014). AS was calculated by computing the difference between MMR and mRMR. To determine S_{crit}, we found the first value where the shark's metabolic rate dropped below mRMR and where the remaining metabolic rates were also below mRMR. Those metabolic rates were isolated, and linear regression was applied to those values and the oxygen content associated with each value (ranged from 2 – 11 values). The oxygen content where the regression line and mRMR intersected was defined as C_{crit} (Schurmann and Steffensen, 1997). S_{crit} was calculated by dividing C_{crit} by the oxygen concentration at 100% saturation at the tested temperature. Lastly, C_{crit} was converted into critical oxygen partial pressure (P_{crit}) by calculating the partial pressure of oxygen at 1 atmosphere for the start day of the trial in Wachapreague, VA, USA and multiplying by S_{crit}.

A multivariate repeated-measures mixed effects model was developed in SAS 9.4 (SAS Institute) using the MIXED procedure to understand the effect of temperature on MMR, SMR, AS, and P_{crit} (Lapointe, et al., 2014). The responses were MMR, SMR, AS, and P_{crit} for each trial, the covariate was temperature, and the random effect was individual fish. To maintain the assumption of normality, the four responses were multiplied by a constant so they would be on the same scale as MMR prior to being put in the model. We modeled the heterogeneity in responses among temperature treatments and specified the Kenward-Roger method for

For Review Only

calculating the degrees of freedom (Kenward and Roger, 1997). Compound Symmetry, AR1, and Toeplitz correlation structures were fitted to the data and the Bayesian Information Criterion (BIC) was used to identify the model with the most supported correlation structure (Littell *et al.*, 2006). Our model chosen for inference included a correlation structure of AR1. Lastly, a priori contrast statements of least-square means were generated using the LSMestimate statement in SAS to assess the effects of temperature on the four response variables. At the completion of the analysis, all model estimates were converted back to scale. All statistics were evaluated at significance levels of $\alpha = 0.05$.

To determine how temperature, hypoxia, and shark activity together impacted metabolic rate, linear mixed effects models were fitted to data from sharks equipped with accelerometers. Separate models were applied to data obtained from the normoxic and hypoxic parts of the trials, with the former corresponding to the part of the trial when the oxygen saturation was 80% or higher, and the latter when the oxygen saturation was below 80%. Potential covariates for the normoxic model included temperature, TBF, TBAA, ODBA, shark total length (TL), and the time since start of trial. The full model was used to assess the need to model heterogeneity and correlation structure. Based on BIC, modeling the heterogeneity in responses among temperature treatments and using a correlation structure of AR1 was supported. A series of models was then developed using different combinations of the covariates mentioned above, and the model chosen for inference was selected using BIC. Potential covariates for the hypoxic model included temperature, oxygen content, TBF, TBAA, ODBA, TL, as well as a calculated binary variable denoted as the crash metric. Since it was assumed that metabolic rate will differ before and after C_{crit} is reached, the crash metric consisted of a 1 for metabolic rate values during the hypoxic part of the trial that occurred prior to the shark reaching C_{crit}, and a 0 for the metabolic rates that

For Review Only

occurred after C_{crit} was reached. Similar to the normoxic model, the decision to model heterogeneity in responses among temperatures and with an AR1 correlation structure was based on the full hypoxic model and ultimately supported through BIC. Multiple models were developed with various covariate combinations and BIC was used for model selection. Predicted metabolic rates for the selected normoxic and hypoxic models were generated using estimated marginal means (Searle *et al.*, 1980). Estimates of uncertainty were generated from 1000 bootstrapped samples (Efron and Tibshirani, 1993). Predicted P_{crit} and associated uncertainty was converted back to C_{crit} and S_{crit}. All linear mixed effects models were fitted using the nlme package in R v.3.4.3 (Pinheiro *et al.*, 2013).

Results

Maximum metabolic rate, minimum routine metabolic rate, aerobic scope, & critical oxygen partial pressure

The multivariate repeated measures mixed effects model was successfully fitted and evaluation of diagnostics (e.g., plots of residuals for each response variable) showed reasonable goodness-of-fit. Although there was high variability in measured MMR, mRMR, and AS among individuals (Fig. 3), model results indicated that differences were evident for these three metrics among temperatures. Statistically significant differences were detected over the three experimental temperatures, primarily between 24 and 28°C and between 24 and 32°C (Fig. 4, Table 2). Specifically, MMR increased by 21% and 42% from 24 to 28°C and 32°C, respectively (Fig. 4A; Table 2), while mRMR increased by 18% from 24 to 28°C, and 45% from 24 to 32°C (Fig. 4B; Table 2). Similar to MMR, AS increased considerably from 24 to 28°C (26%) and 24 to 32°C (39%; Fig. 4C; Table 2). In contrast to the other metrics analyzed, mean P_{crit} only increased by 18% and 19% when comparing 24 and 28 to 32°C, respectively and no significant

For Review Only

differences were detected (Fig. 5A; Table 2). Plots of the raw data for MMR, mRMR, AS, S_{crit}, and C_{crit}, are presented in the supplementary data.

Normoxia

The normoxic model with the most empirical support included ODBA, TBAA, time since chase, and an interaction between TBF and temperature. We concluded that each of these covariates was important in explaining variation in the metabolic rate data (see ΔBIC table in Supplementary Table 1). Predicted metabolic rate increased by 47 ± 6 mg O_2 $kg^{-1}h^{-1}$ for every 0.1 unit increase in ODBA (Fig. 6A), whereas metabolic rate only increased by 5 mg O_2 $kg^{-1}h^{-1}$ for every unit increase in TBAA (Fig. 6B). For every hour increase since the animal was chased, the metabolic rate dropped approximately 3 mg O_2 $kg^{-1}h^{-1}$. The estimated effect in metabolic rate over TBF differed among the levels of temperature considered. That is, for every unit increase in TBF, metabolic rate increased 63 and 142 mg O_2 $kg^{-1}h^{-1}$ for 24 and 28°C, respectively. However, at 32°C metabolic rate actually decreased by 20 mg O_2 $kg^{-1}h^{-1}$ for every unit increase in TBF (Fig. 6C). Lastly, it is important to note that during three trials at 32°C during normoxia, we ~~anecdotally~~ documented periods of time when sharks intermittently stopped swimming, ranging from a few seconds to over an hour.

Hypoxia

The model that provided the most parsimonious description of sandbar shark metabolic rate under hypoxic conditions included TBF, temperature, and an interaction between ODBA and oxygen content (see ΔBIC table in Supplementary Table 2). Predicted metabolic rate increased by 55 ± 20 mg O_2 $kg^{-1}h^{-1}$ for every unit increase in TBF. In hypoxic water, metabolic rate

https://mc.manuscriptcentral.com/conphys

differed among temperatures such that as temperature increased from 24 to 28 to 32°C, metabolic rate increased from 149 ± 9 to 183 ± 6 to 217 ± 13 mg O_2 $kg^{-1}h^{-1}$, respectively. Lastly, as ODBA and oxygen content increased, particularly at higher oxygen concentrations (>3.5 mg l^{-1}), predicted metabolic rate also increased. However, as oxygen decreased, particularly at lower oxygen concentrations (<3.5 mg l^{-1}) and ODBA increased, predicted metabolic rate decreased (Fig. 7). Often during hypoxic conditions (<80% oxygen saturation), sharks displayed a banking behavior where they would swim along the edge of the respirometer with its ventral side facing the side. We also observed during hypoxia, two trials at 24°C, three trials at 28°C, and four trials at 32°C periods of time when sharks either stopped swimming completely (in this case the trial was terminated) or intermittently stopped swimming ~~when oxygen was lower in the respirometer~~.

Discussion

To the best of our knowledge this is the first study to measure AS and P_{crit} in an obligate ram-ventilating elasmobranch. In addition, the size of our respirometer allowed us to measure the metabolic rate and activity of juvenile sandbar sharks across a wide range of sizes (79.5 – 113.5 cm TL). We were also able to use the various activity measurements to better understand environmental thresholds of sandbar sharks. Our findings lead us to suggest that caution should be exercised when using activity to estimate field metabolic rate because, as demonstrated in this study, the correlative relationship between activity and metabolic rate breaks down at high temperatures and low oxygen levels.

Maximum metabolic rate, minimum routine metabolic rate, aerobic scope, & normoxia

For Review Only

389 Referenced model response variables (e.g. mRMR, AS, metabolic rate, etc…) below represent model predictions, while raw values are indicated as such. As expected, mRMR values in the present study were higher than the SMR measurements reported by Dowd et al. (2006) for immobile individuals (91 ± 4 mg O_2 $kg^{-1}h^{-1}$ at 24°C; 125 ± 7 mg O_2 $kg^{-1}h^{-1}$ at 28°C). When compared to other obligate ram-ventilators, such as the tuna species (Korsmeyer and Dewar, 2001), sandbar shark AS was substantially lower. Contributing to this trend may be the significantly smaller gill surface area observed in sandbar sharks compared to other obligate ram ventilating teleosts and elasmobranchs (Emery and Szczepanski, 1986). In addition, with a diet consisting of mostly benthic crustaceans and fishes (Ellis and Musick, 2007), juvenile sandbar sharks may not require a high AS.

399 The variation in raw AS among individual juvenile sandbar sharks over the experimental temperature regime suggests that the potential underlying mechanisms describing AS may differ from individual to individual. For example, some individuals displayed a bell-shaped curve in AS over temperature (e.g. SB28, SB31, SB34), some showed an increase in AS with temperature (e.g. SB03, SB12, SB33), and others displayed very little difference in AS over temperature (e.g. SB02, SB04, SB29) (Fig. 3). Due to this high variability, particularly at 32°C, it is difficult to broadly determine if our data support the OCLTT theory (i.e., maximum AS occurs at the optimal temperature); or if sandbar shark AS increases until lethal temperature limits. In individuals that displayed a bell-shaped curve, maximum AS and thus optimal performance occurred at 28°C (24°C < T_{optAS} < 32°C). Whereas, for those individuals that maximized AS at 32°C, we assume AS would decline above 32°C followed by mortality shortly thereafter. This would suggest that performance or fitness is not optimized at AS for those individual sharks. Clark, et al. (2013) suggested that some species display multiple performances – multiple optima

For Review Only

(MPMO), where there are separate optimal temperatures for different physiological processes (e.g. growth, reproduction, and digestion). However, it is difficult to determine if our results support this theory because the optimal temperatures for those processes are unknown for sandbar sharks. The variability in the data may be the result of differences in activity, physical fitness, adaptation, and physiological processes among individuals. Variation in AS may have also resulted from a potential slight underestimation of MMR in some individuals in an effort to avoid mortalities during or following the chase protocol. Further, because temperature could not be controlled in the holding tank and all individuals were tested in the same order of increasing temperature treatments, the relationships of the measured metrics could potentially be affected. For example, sharks may be more acclimated to the chase protocol or the respirometer for subsequent experiments, which could impact MMR and mRMR (Clark, et al., 2013). Lastly, variation in acclimation time for individuals in particular when exposed to 32°C could have affected metabolic rate metrics. Although we were unable to control these aforementioned conditions, it is possible that they contributed~~attributed~~ to the individual variation in these metrics observed here.

Clearer trends were evident when both metabolic rate and activity were considered. Under normoxic conditions, the expected correlation between metabolic rate and activity (Bouyoucos, et al., 2017, Lear, et al., 2017) broke down at 32°C, where the metabolic rate of sandbar sharks actually slightly declined as activity increased (Fig. 6). At high temperatures, sandbar sharks have to delicately balance the increase in oxygen received from increasing their TBF to improve ram ventilation against~~and~~ the energy expenditure needed for locomotion. This is evident when some sharks actually intermittently stopped swimming for periods of time under normoxic conditions at 32°C. A decline in shark performance at 32°C may be due to the

For Review Only

physiological stress incurred from exercise, acute stress, increased demand for oxygen, and/or decreased oxygen in the water. These stressors are known to cause acidosis and hyperkalemia in fishes (Brill *et al.*, 2008, Cliff and Thurman, 1984, Skomal and Mandelman, 2012). It is well known that acidosis and hyperkalemia impact muscle performance, impairing cardiovascular function and potentially reducing stroke volume at a time when a shark may actually need to increase stroke volume to increase oxygen delivery (Farrell *et al.*, 2009, Norin, et al., 2014).

Critical oxygen partial pressure & hypoxia

Based on the P_{crit} values, juvenile sandbar sharks are not hypoxia tolerant. Regardless of temperature, P_{crit} was above those reported for other teleost and elasmobranch species. For example, striped bass (*Morone saxatilis*), a common fish species in Chesapeake Bay, has an S_{crit} of 35 ± 2% and C_{crit} of 2.5 ± 0.2 mg l^{-1}, at 28°C (Lapointe, et al., 2014), both substantially lower than the S_{crit} (51 ± 2%) and C_{crit} (3.3 ± 0.2 mg l^{-1}) of juvenile sandbar sharks at the same temperature (Figs. 4B & 4C). When compared to elasmobranch fishes where P_{crit} was measured, such as the epaulette shark (*Hemiscyllium ocellatum*, 38 ± 3 mmHg) and shovelnose ray (*Aptychotrema rostrate*, 54 ± 3 mmHg), the juvenile sandbar shark's P_{crit} values are likewise substantially higher (Speers-Roesch *et al.*, 2012). When compared to another obligate ram-ventilating species, such as the yellowfin tuna at 25°C (hypoxia tolerance of 3.7 mg l^{-1}; Bushnell & Brill 1991, Bernal et al. 2017), sandbar sharks have a similar hypoxia tolerance. The high P_{crit} values in juvenile sandbar sharks are likely the result of this species also being (like many carcharhinid species) an obligate ram ventilator (Carlson *et al.*, 2004, Dowd, et al., 2006). In hypoxia, obligate ram ventilator species rely on increasing their swimming speed or mouth gape to counteract a decline in oxygen in the water (Bushnell and Brill, 1991, Carlson and Parsons,

For Review Only

2001, Dizon, 1977); however, once P_{crit} is reached, obligate ram-ventilating fishes will not be able to maintain minimum swimming speeds to ~~adequately~~ ventilate their gills adequately or to maintain hydrostatic equilibrium (Carlson and Parsons, 2001). Although, P_{crit} did not differ substantially among temperatures, the mean P_{crit} at 32°C was highest when compared to means at 24 and 28°C, which was expected because as temperature increases, the demand for oxygen increases and the solubility of oxygen in seawater decreases (Schurmann and Steffensen, 1997). The lack of a substantial difference was the result of a high amount variability in P_{crit} values, particularly at 32°C (Fig. 5A). Sandbar shark metabolic rate did increase as temperature increased in hypoxic waters (<80% oxygen saturation), suggesting that at warmer temperatures (~32°C), the demand for oxygen is higher while in less oxygenated water, which can lead to increased stress and a higher risk of mortality. An alternative hypothesis is that juvenile sandbar sharks are adapted to large tidal fluctuations in temperature (4.6°C; D. Kelley unpublished data); therefore, the ability to deliver oxygen to the tissues is not compromised due to increased temperatures.

In hypoxic waters, sandbar shark metabolic rate was highly dependent on oxygen level and the shark's activity. The synergistic effect of oxygen and activity on metabolic rate led to~~displayed~~ different trends above and below approximately 3.5 mg l^{-1}, which was similar to the mean (± SE) C_{crit} of sandbar sharks between 24 - 32°C (3.4 ± 0.1 mg l^{-1}). The positive correlation of metabolic rate and activity, as oxygen concentrations increased from 3.5 mg l^{-1}, followed trends similar to those when sharks were under normoxic conditions (>80% oxygen saturation). When oxygen dropped below 3.5 mg l^{-1}, however, an inverse relationship occurred between metabolic rate and activity, a trend that was also evident at 32°C under normoxic conditions. The breakdown in the positive relationship between metabolic rate and activity suggests that at these

For Review Only

lower oxygen levels sandbar sharks employ anaerobic metabolism to power swimming, which ~~in turn, again,~~ can lead to acidosis and hyperkalemia. It is clear this behavior cannot be sustained, based on the many periods of time when sharks stopped swimming either completely or intermittently during hypoxia. According to our data~~,~~ the oxygen concentration threshold for juvenile sandbar sharks is approximately 3.5 mg l^{-1} and ~~that~~ this threshold is positively correlated with temperature.

Climate Change Impacts

It is critical to understand environmental thresholds for marine species amidst climate change. Although sandbar sharks are not expected to encounter 32°C waters often, the potential to encounter these temperatures will increase as climate change continues to alter marine environments, particularly within coastal habitats. With an increase in temperature we also expect to see an increase in the extent and severity of hypoxic waters which, may have a large impact on the obligate ram ventilating sandbar shark. For example, along the eastern shore of VA, where known sandbar shark nursery habitat exists, water temperatures during the months of July and August during 2016 and 2017 exceeded 32°C at times (range: 21.3 – 32.8°C; mean: 27.4°C; D. Kelley unpublished data) and oxygen levels ranged from 2.9 – 8.2 mg l^{-1}. By the mid-21st century, Chesapeake Bay, which is the largest sandbar shark nursery habitat in the United States, is expected to increase 1.75°C relative to the mid-1990s (Muhling *et al.*, 2018). In addition, by the end of the century, heat waves are also projected to increase by over two standard deviations along the Mid-Atlantic (Najjar, et al., 2010). Also by 2050, Chesapeake Bay is predicted to have the largest increase in cumulative hypoxic volume (72 - 202 km^3 days) at oxygen concentrations between 2 - 5 mg l^{-1} (Irby et al. 2018), a range of oxygen concentrations

For Review Only

that include the P_{crit} of juvenile sandbar sharks. This volume of hypoxic water is expected to occur earlier in the summer as well (Irby, et al., 2018). These environmental changes will likely significantly diminish the suitability of Chesapeake Bay and adjacent coastal areas to serve as nursery habitat for sandbar sharks in the western Atlantic. As climate change impacts worsen, juvenile sandbar sharks, which can spend up to 10 years in these nursery habitats, may see available habitat reduced and be forced to seek out novel nursery habitats or risk increases in juvenile mortality. This may, in turn, affect the overall abundance of an already overfished sandbar shark population (SEDAR, 2017). At an ecological level, juvenile sandbar sharks are top predators within coastal habitats and may control the populations of other fish species (Ellis and Musick, 2007). Shifts in juvenile sandbar shark distribution could, therefore, also have negative effects on the population of lower trophic species within these habitats.

Conclusions

This study was able to identify the temperature and oxygen thresholds of juvenile sandbar sharks. We found that their performance substantially declines at 32°C (even in normoxia) and at oxygen concentrations below 3.5 mg l^{-1}, and that activity becomes inversely correlated with oxygen consumption rates under high stress conditions. These impacts suggest that in the face of climate change, areas of Chesapeake Bay may become less suitable nursery habitat for sandbar sharks. It is critical for future studies to use environmental thresholds like those identified in this study to predict species distribution under climate change scenarios to understand potential habitat shifts.

For Review Only

Tables

Table 1. Size and treatment summary of the juvenile sandbar sharks in the study.

Animal ID	Year Tested	Animal Mass (kg)	Total Length (cm)	Treatments (°C)	Accelerometer
SB02	2016	7.30	109	24, 28, 32	No
SB03	2016	2.66	80	24, 28, 32	No
SB04	2016	5.09	99	24, 28, 32	No
SB05	2016	5.46	98	24, 28, 32	No
SB12	2016	5.69	103.5	24, 28, 32	No
SB28	2017	7.76	113.5	24, 28, 32	Yes
SB29	2017	3.31	91	24, 28, 32	Yes
SB30	2017	4.22	94	24, 28, 32	Yes
SB31	2017	3.98	91	24, 28, 32	Yes
SB32	2017	2.56	81	24, 28	Yes
SB33	2017	6.81	109	24, 28, 32	Yes
SB34	2017	6.84	107	24, 28, 32	Yes
SB37	2017	2.60	79.5	32	Yes

Table 2. T statistics from the a priori contrast statements of least-square means generated from the multivariate repeated measures mixed effects model to assess the impact of temperature (24, 28, and 32°C) on MMR, mRMR, AS and P_{crit}. The degrees of freedom are in parentheses. The (*) signifies a significant difference between the two temperatures.

Metric	24°C x 28°C	24°C x 32°C	28°C x 32°C
MMR	$t_{(57.34)} = -2.33$*	$t_{(47.03)} = -3.12$*	$t_{(56.82)} = -1.43$
mRMR	$t_{(57.34)} = -1.90$	$t_{(47.03)} = -3.25$*	($t_{(56.11)} = -1.81$
AS	$t_{(57.34)} = -2.81$*	$t_{(47.03)} = -2.90$*	$t_{(56.11)} = -0.93$
P_{crit}	$t_{(56.82)} = 0.14$	$t_{(46.93)} = -1.29$	$t_{(59.02)} = -1.25$

For Review Only

Figures

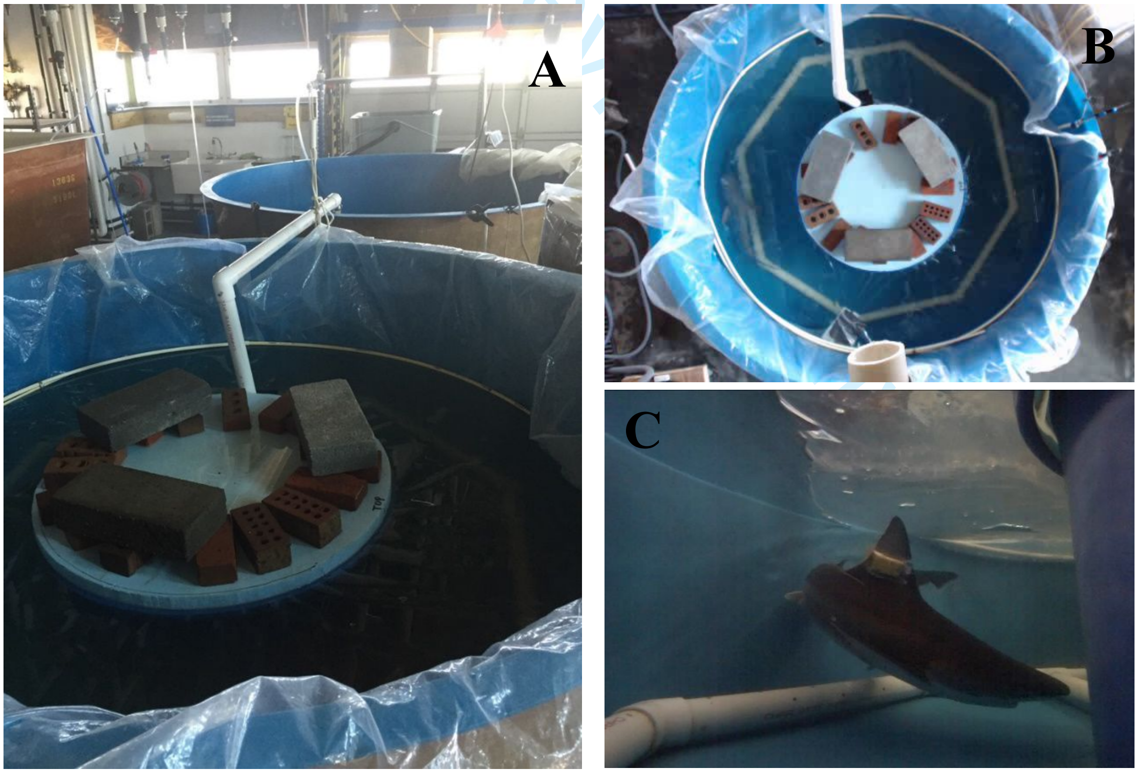

Figure 1. (A) The sandbar shark respirometer system with the respirometer in the foreground and the reservoir in the background. A PVC pipe connects the flush pump to the diffuser in the bottom of the respirometer. (B) An overhead view of the respirometer with a shark present. The

PVC octagon is the diffuser that ensures mixing during the flush cycle. (C) A shark in the respirometer with an accelerometer fitted to its dorsal fin.

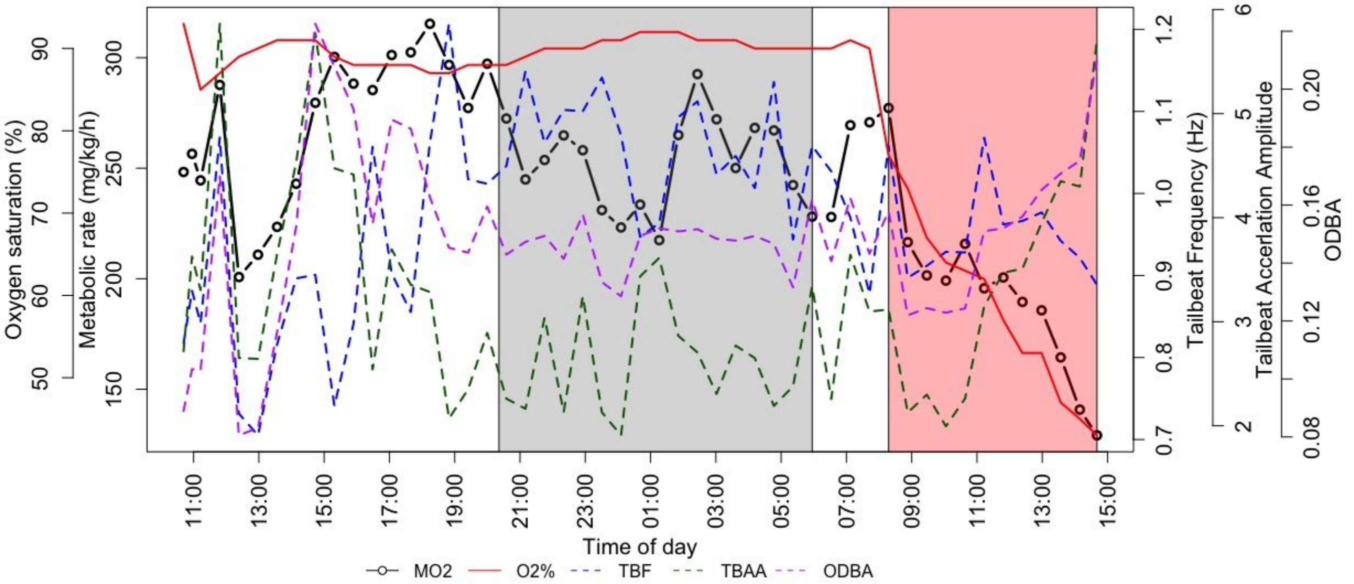

Figure 2. An example of a full trial from SB33 tested at 28°C. Each point corresponds to a calculated metabolic rate (MO_2) along with the associated oxygen saturation (O_2%), tailbeat frequency (TBF), tailbeat acceleration amplitude (TBAA), and ODBA values. The grey shaded area represents night hours created from sunrise/sunset time. The red shaded area represents the hypoxia period (O_2% < 80%). S_{crit} for this individual at 28°C was 58% oxygen saturation.

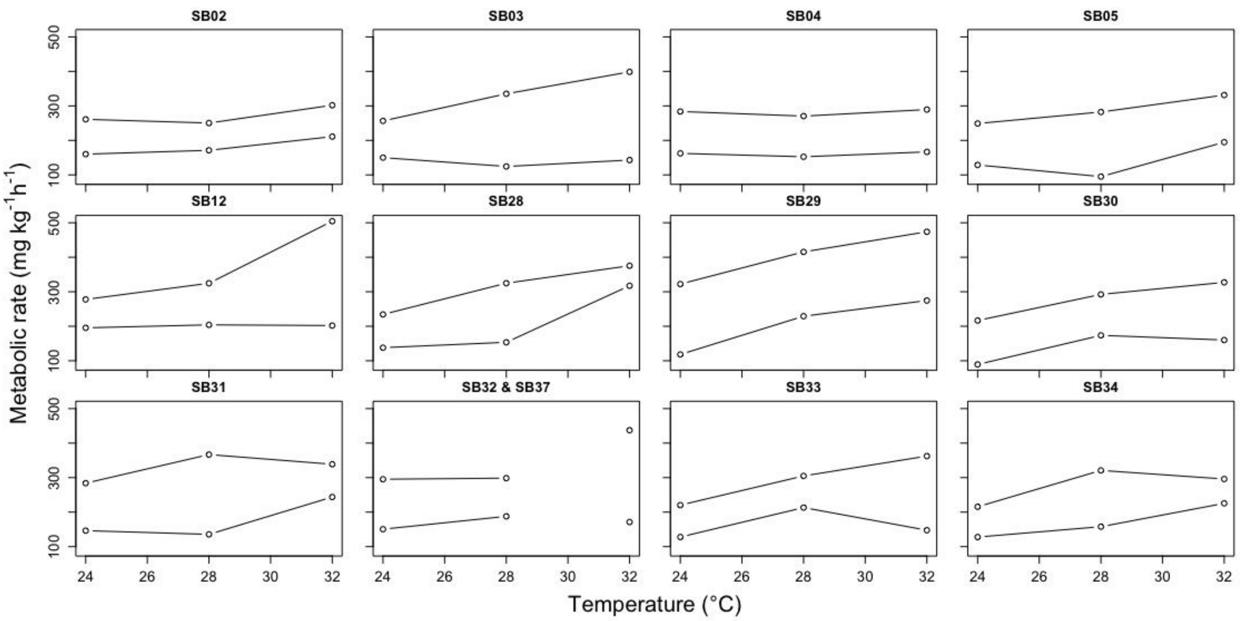

Figure 3. Measured maximum metabolic rate (MMR; top line in each plot) and minimum routine metabolic rate (mRMR; bottom line in each plot) for each shark tested in this study. The metabolic rate was measured at 24, 28, and 32°C for each shark except for SB32 which was only measured at 24 and 28°C (line connected those two data points). SB37 replaced SB32 at 32°C (single points).

Review Only

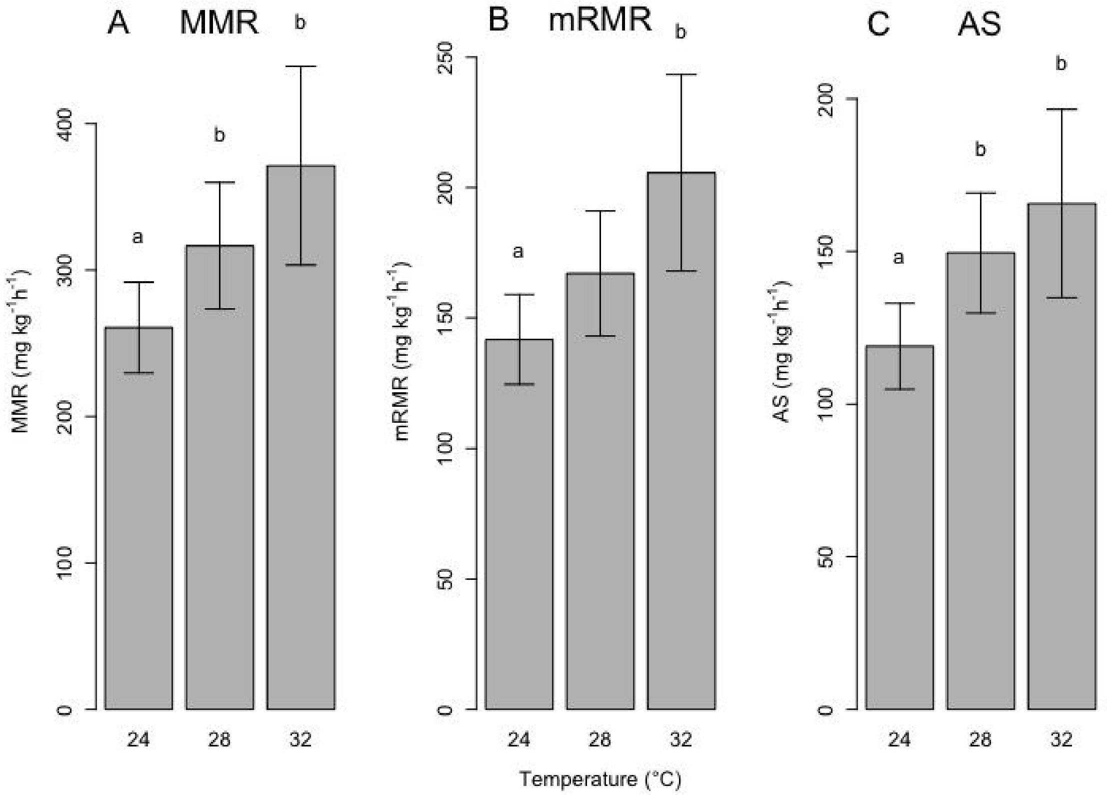

Figure 4. (A) Maximum metabolic rate (MMR), (B) minimum routine metabolic rate (mRMR), and (C) aerobic scope (AS) at 24, 28, and 32°C of sandbar sharks when in normoxic waters. Data are model estimates of the mean ± 95% CI. Different lower case letters indicate a significant difference between two temperatures, whereas the lack of a letter indicates no significance.

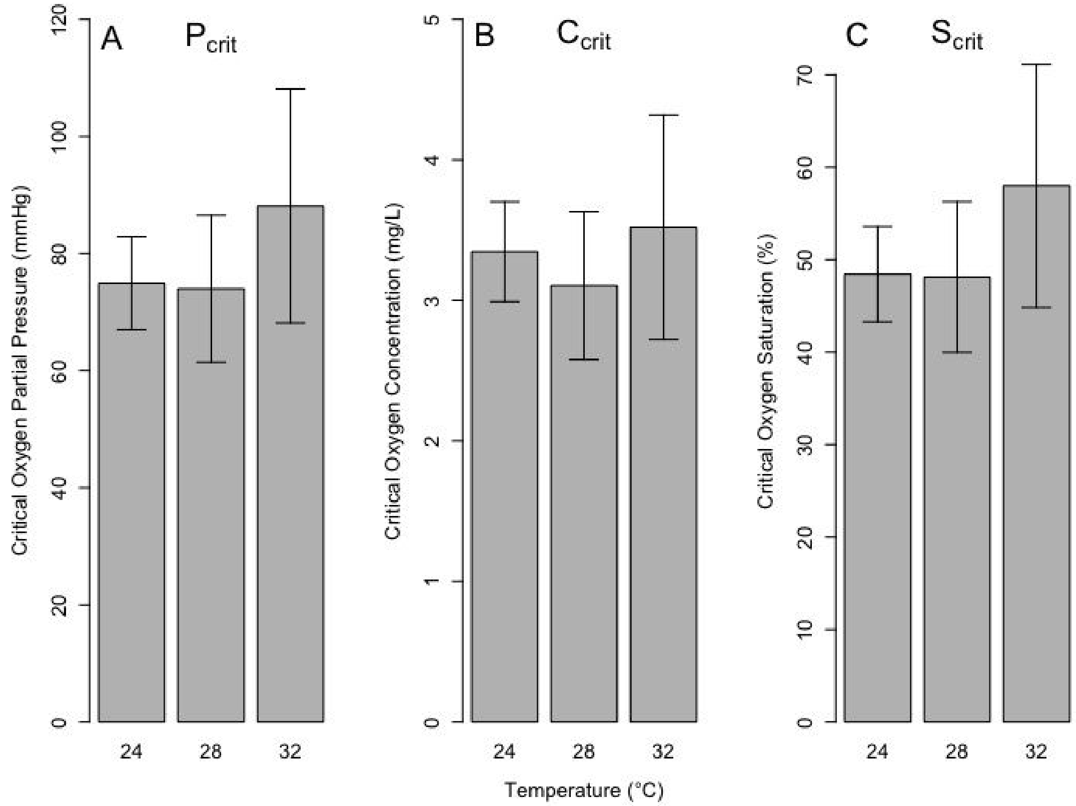

Figure 5. (A) Critical oxygen partial pressure (P_{crit}), (B) critical oxygen concentration (C_{crit}), and (C) critical oxygen saturation (S_{crit}) at 24, 28, and 32°C of sandbar sharks when in normoxic waters. P_{crit} data are model estimates of the mean ± 95% CI. C_{crit} and S_{crit} were calculated from P_{crit} model estimates. The lack of a letter indicates no significance.

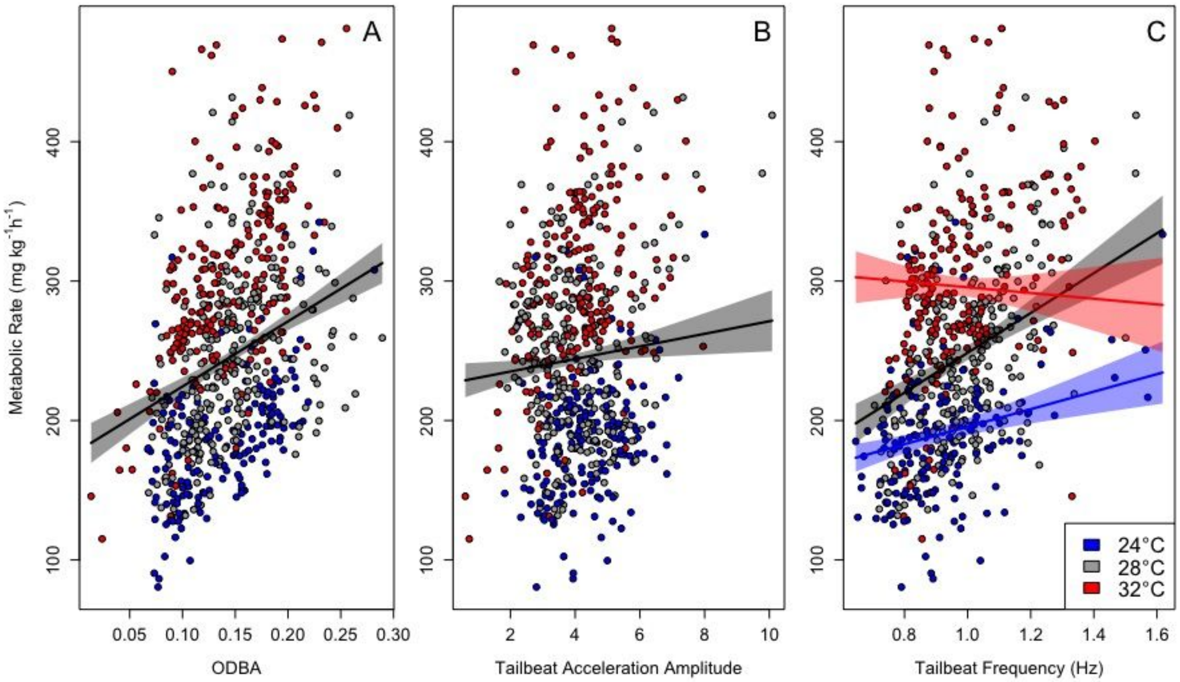

655
656 Figure 6. Metabolic rate values by ODBA (A), tailbeat acceleration amplitude (B), and tailbeat
657 frequency (C) when sandbar sharks are exposed to normoxic waters. The colored circles
658 represent the observed metabolic rate values for a given temperature. The line and associated
659 shaded region represent the estimated metabolic rate values and the 95% CI, respectively. An
660 interaction did not occur between temperature and ODBA (A) or tailbeat acceleration amplitude
661 (B); therefore, temperature was combined when estimating metabolic rate for those metrics.
662 However, because there was a significant interaction between temperature and tailbeat frequency
663 there are separate estimated metabolic rates for each temperature which are represented by
664 different colored lines and shaded regions in C.
665
666
667

Review Only

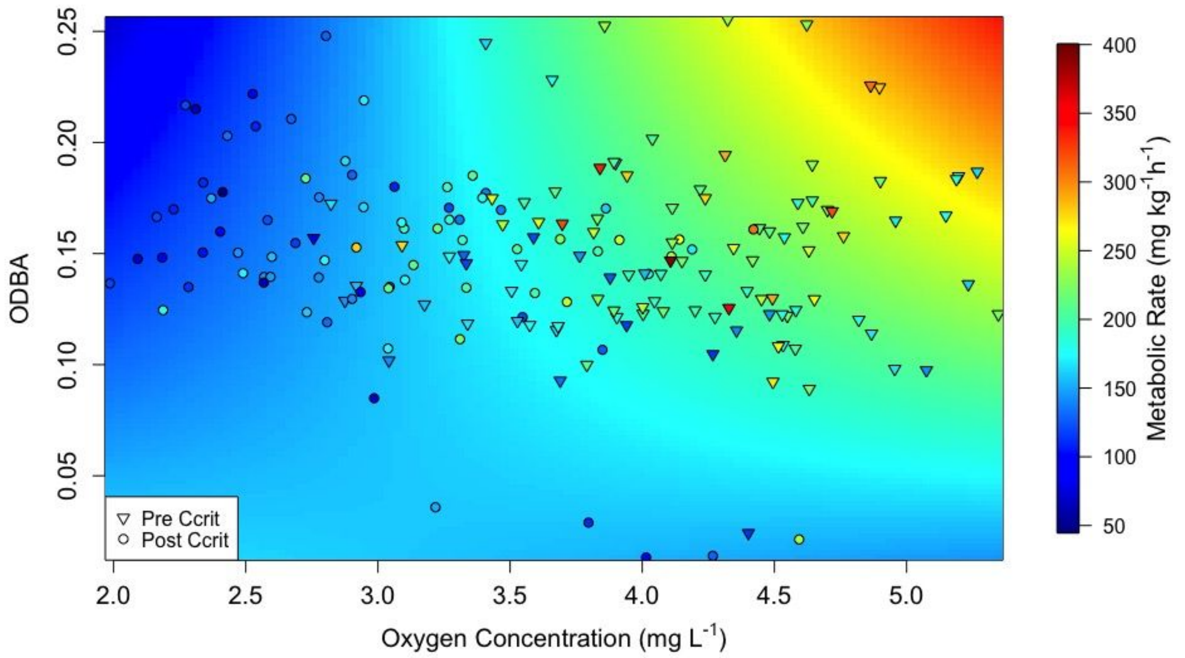

Figure 7. Metabolic rate in relation to ODBA and oxygen concentration during hypoxia (< 80% oxygen saturation). Symbols represent observed data, where ∇ are before C_{crit} was reached and O are after C_{crit} was reached. The colors on the symbols represent the observed metabolic rate. The surface behind the symbols represents the predicted metabolic rate estimated from the model.

Supplementary Data

Supplementary Table 1. ΔBIC table of potential models to describe metabolic rate under normoxic conditions. All of these models are already assumed to include heterogeneity and a correlation structure of AR1.

Model #	Models describing metabolic rate	DF	ΔBIC
1	(1|Animal_ID) + ODBA + (TBF * Temp) + TBA + Trial_Time	14	0
2	(1|Animal_ID) + (ODBA * Temp) + (TBF * Temp) + TBA + Trial_Time	16	11.1
3	(1|Animal_ID) + ODBA + TBF + Temp + Trial_Time	11	23.1
4	(1|Animal_ID) + (ODBA * Temp) + (TBF * Temp) + (TBA * Temp) + Trial_Time	18	23.4
5	(1|Animal_ID) + (ODBA * Temp) + TBF + TBA + Trial_Time	14	24.4
6	(1|Animal_ID) + ODBA + TBF + TBA + Temp + Trial_Time	12	26.6
7	(1|Animal_ID) + ODBA + TBF + Temp + TL + Trial_Time	12	29.3
8	(1|Animal_ID) + ODBA + TBF + TBA + Temp + TL + Trial_Time	13	32.9
9	(1|Animal_ID) + ODBA + TBF + Temp + Trial_Time	10	68.2

Supplementary Table 2. ΔBIC table of potential models to describe metabolic rate under hypoxic conditions. All of these models are already assumed to include heterogeneity and a correlation structure of AR1.

Model #	Models describing metabolic rate	DF	ΔBIC
1	(1|Animal_ID) + (ODBA * O2) + TBF + Temp	12	0
2	(1|Animal_ID) + (ODBA * O2) + TBF + TBA + Temp	13	2.2
3	(1|Animal_ID) + (ODBA * O2) + (TBF * O2) + Temp	13	3.9
4	(1|Animal_ID) + (ODBA * O2) + TBF + TL + Temp	13	4.4
5	(1|Animal_ID) + (ODBA * O2) + TBF + TBA + TL + Temp	14	6.6
6	(1|Animal_ID) + (ODBA * O2) + (TBF * O2) + TBA + TL + Temp	15	9.6
7	(1|Animal_ID) + (ODBA * O2) + (TBF * O2) + (ODBA * Temp)	15	12.4
8	(1|Animal_ID) + (ODBA * O2) + (TBF * O2) + (ODBA * Temp) + TL	16	16.8
9	(1|Animal_ID) + (ODBA * O2) + (TBF * O2) + (ODBA * Temp) + TBA + TL	17	18.5
10	(1|Animal_ID) + ODBA + TBF + TBA + Temp + O2 + TL	13	21.9
11	(1|Animal_ID) + ODBA + TBF + TBA + Temp + Crash_metric + TL	13	76.4

For Review Only

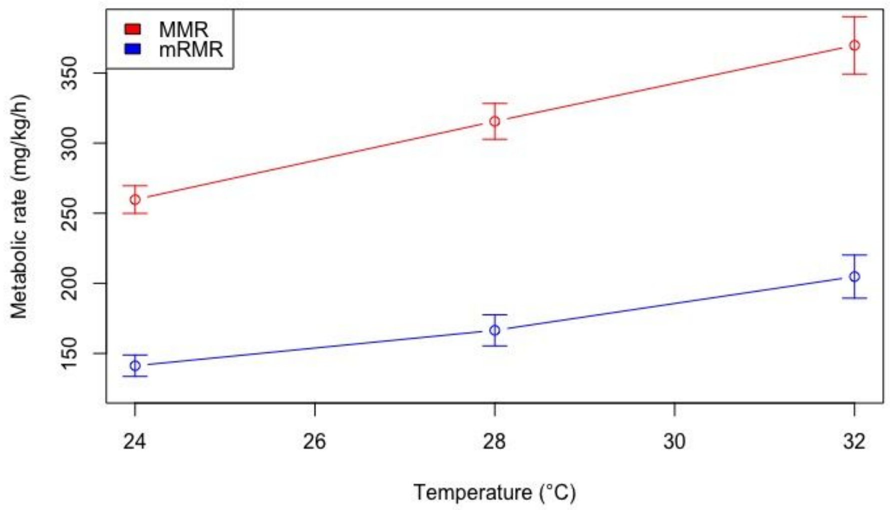

Supplementary Figure 1. Raw means (± SE) of maximum metabolic rate (MMR) and minimum routine metabolic rate (mRMR) of sandbar sharks tested at 24, 28, and 32°C.

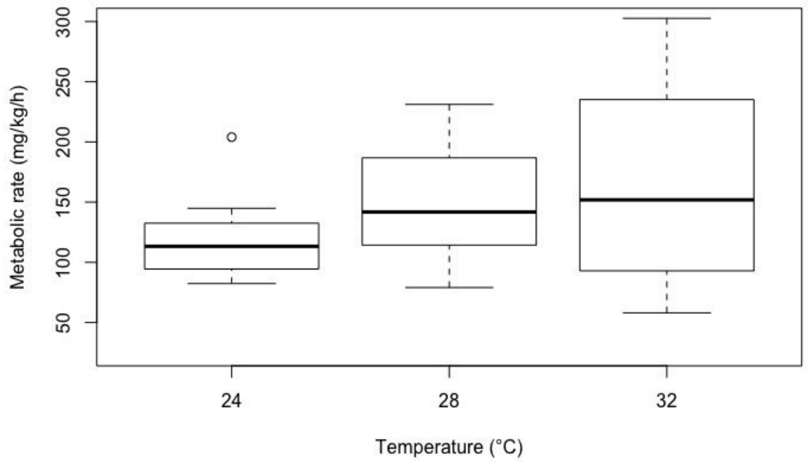

Supplementary Figure 2. Raw values of aerobic scope of sandbar sharks tested at 24, 28, and 32°C.

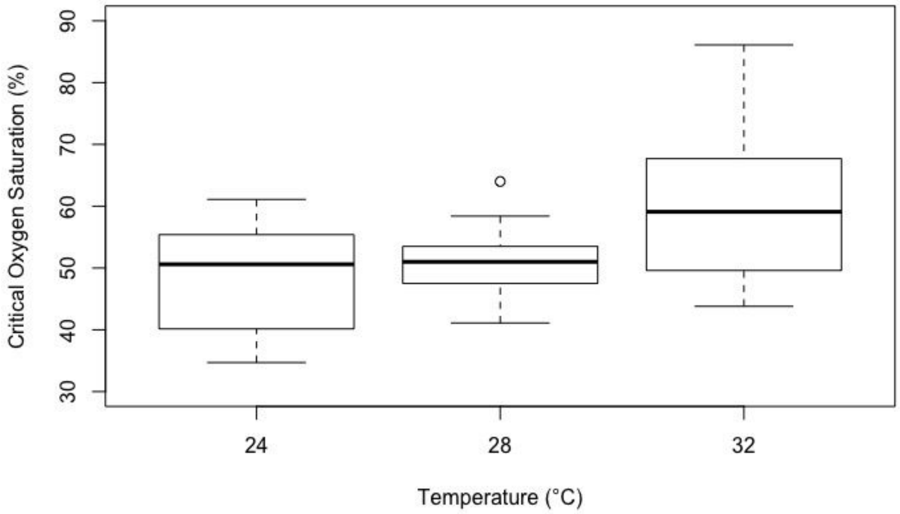

Supplementary Figure 3. Raw values of the critical oxygen saturation (S_{crit}) of sandbar sharks tested at 24, 28, and 32°C.

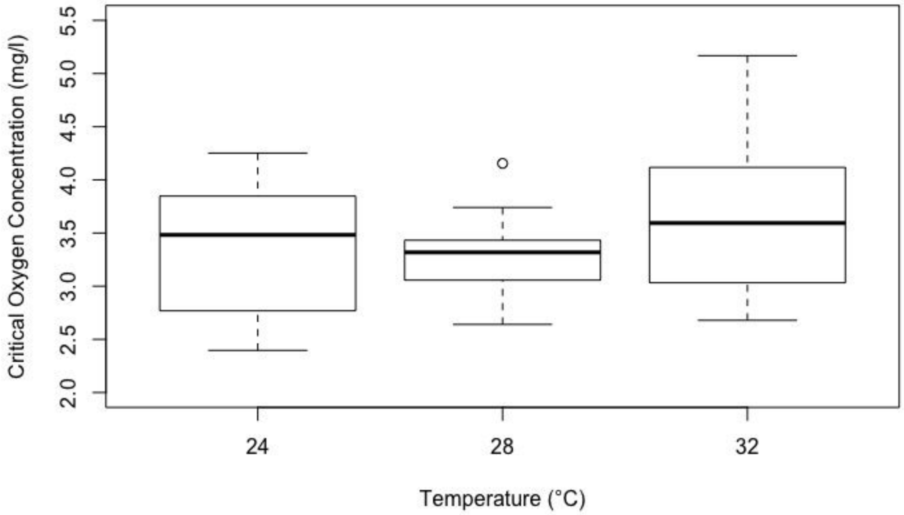

Supplementary Figure 4. Raw values of the critical oxygen concentration (C_{crit}) of sandbar sharks tested at 24, 28, and 32°C.

Funding

This work was supported by Virginia Institute of Marine Science, College of William & Mary.

Acknowledgements

We thank the staff and interns at the Virginia Institute of Marine Science Eastern Shore Laboratory, particularly Chris Bentley, Stephanie Bonniewell, and Sean Fate for their help in animal collection, guidance on animal husbandry, and providing shark holding and laboratory facilities. We also thank Mary Fabrizio and Cassidy Peterson for their statistical advice.

For Review Only

References

Bangley CW, Paramore L, Shiffman DS, Rulifson RA (2018) Increased Abundance and Nursery Habitat Use of the Bull Shark (*Carcharhinus leucas*) in Response to a Changing Environment in a Warm-Temperate Estuary. *Scientific Reports* 8: 6018

Bernal D, Brill RW, Dickson KA, Shiels HAJRiFB, Fisheries (2017) Sharing the water column: physiological mechanisms underlying species-specific habitat use in tunas 27: 843-880

Bouyoucos IA, Montgomery DW, Brownscombe JW, Cooke SJ, Suski CD, Mandelman JW, Brooks EJ (2017) Swimming speeds and metabolic rates of semi-captive juvenile lemon sharks (*Negaprion brevirostris*, Poey) estimated with acceleration biologgers. *Journal of Experimental Marine Biology and Ecology* 486: 245-254

Brill R, Bushnell P, Schroff S, Seifert R, Galvin M (2008) Effects of anaerobic exercise accompanying catch-and-release fishing on blood-oxygen affinity of the sandbar shark (*Carcharhinus plumbeus*, Nardo). *Journal of Experimental Marine Biology and Ecology* 354: 132-143

Brill RW, Bushnell PG, Elton TA, Small HJ (2015) The ability of blue crab (*Callinectes sapidus*, Rathbun 1886) to sustain aerobic metabolism during hypoxia. *Journal of Experimental Marine Biology and Ecology* 471: 126-136

Bushnell PG, Brill RW (1991) Responses of swimming skipjack (*Katsuwonus pelamis*) and yellowfin (*Thunnus albacares*) tunas to acute hypoxia, and a model of their cardiorespiratory function. *Physiological Zoology* 64: 787-811

Carlson JK, Goldman KJ, Lowe CG (2004) Metabolism, energetic demand, and endothermy. In Carrier J, Musick J, Heithaus M eds, Biology of sharks and their relatives Vol. 10, pp 269-286

Carlson JK, Parsons GR (2001) The effects of hypoxia on three sympatric shark species: physiological and behavioral responses. *Environmental Biology of Fishes* 61: 427-433

Claireaux G, Lefrançois C (2007) Linking environmental variability and fish performance: integration through the concept of scope for activity. *Philosophical Transactions of the Royal Society B: Biological Sciences* 362: 2031-2041

Clark TD, Jeffries KM, Hinch SG, Farrell AP (2011) Exceptional aerobic scope and cardiovascular performance of pink salmon (*Oncorhynchus gorbuscha*) may underlie resilience in a warming climate. *The Journal of Experimental Biology* 214: 3074-3081

For Review Only

Clark TD, Sandblom E, Jutfelt F (2013) Aerobic scope measurements of fishes in an era of climate change: respirometry, relevance and recommendations. *Journal of Experimental Biology* 216: 2771-2782

Cliff G, Thurman G (1984) Pathological and physiological effects of stress during capture and transport in the juvenile dusky shark, Carcharhinus obscurus. *Comparative Biochemistry and Physiology Part A: Physiology* 78: 167-173

Conley DJ, Carstensen J, Ærtebjerg G, Christensen PB, Dalsgaard T, Hansen JL, Josefson AB (2007) Long-term changes and impacts of hypoxia in Danish coastal waters. *Ecological Applications* 17: S165-S184

Conrath CL, Musick JA (2007) The sandbar shark summer nursery within bays and lagoons of the eastern shore of Virginia. *Transactions of the American Fisheries Society* 136: 999-1007

Di Santo V (2016) Intraspecific variation in physiological performance of a benthic elasmobranch challenged by ocean acidification and warming. *Journal of Experimental Biology* 219: 1725-1733

Diaz RJ, Rosenberg R (1995) Marine benthic hypoxia: a review of its ecological effects and the behavioural responses of benthic macrofauna. In Ansell A, Gibson R, Barnes M eds, Oceanography and Marine Biology. An Annual Review Vol. 33, London, UK: University College London Press, pp 245-303

Dizon A (1977) Effect of dissolved oxygen concentrations and salinity on swimming speed of two species of tuna. *Fisheries Bulletin* 75: 649-653

Dowd WW, Brill RW, Bushnell PG, Musick JA (2006) Standard and routine metabolic rates of juvenile sandbar sharks (*Carcharhinus plumbeus*), including the effects of body mass and acute temperature change. *Fishery Bulletin* 104: 323-331

Efron B, Tibshirani RJ (1993) An introduction to the bootstrap. CRC press, New York, NY.

Ellis JK, Musick JA (2007) Ontogenetic changes in the diet of the sandbar shark, *Carcharhinus plumbeus*, in lower Chesapeake Bay and Virginia (USA) coastal waters. *Environmental Biology of Fishes* 80: 51-67

Emery SH, Szczepanski A (1986) Gill dimensions in pelagic elasmobranch fishes. *The Biological Bulletin* 171: 441-449

Farrell A, Eliason E, Sandblom E, Clark T (2009) Fish cardiorespiratory physiology in an era of climate change. *Canadian Journal of Zoology* 87: 835-851

For Review Only

Fry FEJ (1971) The effect of environmental factors on the physiology of fish. *Fish Physiology* 6: 1-98

Fry FEJ, Hart J (1948) The relation of temperature to oxygen consumption in the goldfish. *The Biological Bulletin* 94: 66-77

Gleiss AC, Wilson RP, Shepard EL (2011) Making overall dynamic body acceleration work: on the theory of acceleration as a proxy for energy expenditure. *Methods in Ecology and Evolution* 2: 23-33

Grubbs RD, Musick J (2007). Spatial delineation of summer nursery areas for juvenile sandbar sharks in Chesapeake Bay, Virginia, American Fisheries Society Symposium Vol. 50, American Fisheries Society, pp 63-86

Hagy JD, Boynton WR, Keefe CW, Wood KV (2004) Hypoxia in Chesapeake Bay, 1950–2001: long-term change in relation to nutrient loading and river flow. *Estuaries* 27: 634-658

Irby ID, Friedrichs MA, Da F, Hinson KE (2018) The competing impacts of climate change and nutrient reductions on dissolved oxygen in Chesapeake Bay. *Biogeosciences* 15: 2649-2668

Irby ID, Friedrichs MA, Friedrichs CT, Bever AJ, Hood RR, Lanerolle LW, Scully ME (2016) Challenges associated with modeling low-oxygen waters in Chesapeake Bay: a multiple model comparison. *Biogeosciences* 13: 2011-2028

Kemp WM, Boynton WR, Adolf JE, Boesch DF, Boicourt WC, Brush G, Cornwell JC, Fisher TR, Glibert PM, Hagy JD (2005) Eutrophication of Chesapeake Bay: historical trends and ecological interactions. *Marine Ecology Progress Series* 303: 1-29

Kenward MG, Roger JH (1997) Small sample inference for fixed effects from restricted maximum likelihood. *Biometrics*: 983-997

Killen SS, Norin T, Halsey LG (2017) Do method and species lifestyle affect measures of maximum metabolic rate in fishes? *Journal of Fish Biology* 90: 1037-1046

Korsmeyer K, Dewar H (2001) Tuna metabolism and energetics. Tuna physiology, ecology and evolution. In Block B, Stevens E eds, Fish Physiology Vol. 19, San Diego, CA: Academic Press, pp 35-78

Lapointe D, Vogelbein WK, Fabrizio MC, Gauthier DT, Brill RW (2014) Temperature, hypoxia, and mycobacteriosis: effects on adult striped bass Morone saxatilis metabolic performance. *Dis. Aquat. Org* 108: 113-127

For Review Only

Last PR, White WT, Gledhill DC, Hobday AJ, Brown R, Edgar GJ, Pecl G (2011) Long-term shifts in abundance and distribution of a temperate fish fauna: a response to climate change and fishing practices. *Global Ecology and Biogeography* 20: 58-72

Lear KO, Whitney NM, Brewster LR, Morris JJ, Hueter RE, Gleiss AC (2017) Correlations of metabolic rate and body acceleration in three species of coastal sharks under contrasting temperature regimes. *Journal of Experimental Biology* 220: 397-407

Lefevre S (2016) Are global warming and ocean acidification conspiring against marine ectotherms? A meta-analysis of the respiratory effects of elevated temperature, high CO2 and their interaction. *Conservation Physiology* 4: 1-31

Littell RC, Milliken GA, Stroup WW, Wolfinger RD, Schabenberger O (2006) SAS for mixed models, 2nd edn, SAS Inst., Cary, NC.

Ludsin SA, Zhang X, Brandt SB, Roman MR, Boicourt WC, Mason DM, Costantini M (2009) Hypoxia-avoidance by planktivorous fish in Chesapeake Bay: implications for food web interactions and fish recruitment. *Journal of Experimental Marine Biology and Ecology* 381: S121-S131

Lynch PD, Nye JA, Hare JA, Stock CA, Alexander MA, Scott JD, Curti KL, Drew K (2014) Projected ocean warming creates a conservation challenge for river herring populations. *ICES Journal of Marine Science* 72: 374-387

McKenzie DJ, Axelsson M, Chabot D, Claireaux G, Cooke SJ, Corner RA, De Boeck G, Domenici P, Guerreiro PM, Hamer B (2016) Conservation physiology of marine fishes: state of the art and prospects for policy. *Conservation Physiology* 4: 1-20

Morley JW, Selden RL, Latour RJ, Frölicher TL, Seagraves RJ, Pinsky ML (2018) Projecting shifts in thermal habitat for 686 species on the North American continental shelf. *PloS one* 13: 1-28

Muhling BA, Gaitán CF, Stock CA, Saba VS, Tommasi D, Dixon KW (2018) Potential salinity and temperature futures for the Chesapeake Bay using a statistical downscaling spatial disaggregation framework. *Estuaries and Coasts* 41: 349-372

Najjar RG, Pyke CR, Adams MB, Breitburg D, Hershner C, Kemp M, Howarth R, Mulholland MR, Paolisso M, Secor D (2010) Potential climate-change impacts on the Chesapeake Bay. *Estuarine, Coastal and Shelf Science* 86: 1-20

For Review Only

Norin T, Malte H, Clark TD (2014) Aerobic scope does not predict the performance of a tropical eurythermal fish at elevated temperatures. *Journal of Experimental Biology* 217: 244-251

Nye JA, Link JS, Hare JA, Overholtz WJ (2009) Changing spatial distribution of fish stocks in relation to climate and population size on the Northeast United States continental shelf. *Marine Ecology Progress Series* 393: 111-129

Payne NL, Meyer CG, Smith JA, Houghton JD, Barnett A, Holmes BJ, Nakamura I, Papastamatiou YP, Royer MA, Coffey DM (2018) Combining abundance and performance data reveals how temperature regulates coastal occurrences and activity of a roaming apex predator. *Global Change Biology*: 1-10

Payne NL, Smith JA, Meulen DE, Taylor MD, Watanabe YY, Takahashi A, Marzullo TA, Gray CA, Cadiou G, Suthers IM (2016) Temperature dependence of fish performance in the wild: links with species biogeography and physiological thermal tolerance. *Functional Ecology* 30: 903-912

Pinheiro J, Bates D, DebRoy S, Sarkar D (2013) nlme: Linear and Nonlinear Mixed Effects Models. R package version 3.1-111 <http://CRAN.R-project.org/package=nlme>

Pörtner HO, Knust R (2007) Climate change affects marine fishes through the oxygen limitation of thermal tolerance. *Science* 315: 95-97

Preston BL (2004) Observed winter warming of the Chesapeake Bay estuary (1949–2002): implications for ecosystem management. *Environmental Management* 34: 125-139

Rabalais NN, Turner RE, Díaz RJ, Justić D (2009) Global change and eutrophication of coastal waters. *ICES Journal of Marine Science: Journal du Conseil* 66: 1528-1537

Rijnsdorp AD, Peck MA, Engelhard GH, Möllmann C, Pinnegar JK (2009) Resolving the effect of climate change on fish populations. *ICES Journal of Marine Science: Journal du Conseil*: 1-14

Schurmann H, Steffensen J (1997) Effects of temperature, hypoxia and activity on the metabolism of juvenile Atlantic cod. *Journal of Fish Biology* 50: 1166-1180

Searle SR, Speed FM, Milliken GAJTAS (1980) Population marginal means in the linear model: an alternative to least squares means 34: 216-221

SEDAR (2017). SEDAR 54 stock assessment report: HMS Sandbar Shark, North Charleston, SC

For Review Only

Shepard EL, Wilson RP, Quintana F, Laich AG, Liebsch N, Albareda DA, Halsey LG, Gleiss A, Morgan DT, Myers AE (2008) Identification of animal movement patterns using tri-axial accelerometry. *Endangered Species Research* 10: 47-60

Skomal GB, Mandelman JW (2012) The physiological response to anthropogenic stressors in marine elasmobranch fishes: a review with a focus on the secondary response. *Comparative Biochemistry and Physiology Part A: Molecular & Integrative Physiology* 162: 146-155

Speers-Roesch B, Richards JG, Brauner CJ, Farrell AP, Hickey AJ, Wang YS, Renshaw GM (2012) Hypoxia tolerance in elasmobranchs. I. Critical oxygen tension as a measure of blood oxygen transport during hypoxia exposure. *Journal of Experimental Biology* 215: 93-102

Svendsen M, Bushnell P, Steffensen J (2016) Design and setup of intermittent-flow respirometry system for aquatic organisms. *Journal of Fish Biology* 88: 26-50

Turner SM, Manderson JP, Richardson DE, Hoey JJ, Hare JA (2015) Using habitat association models to predict Alewife and Blueback Herring marine distributions and overlap with Atlantic Herring and Atlantic Mackerel: can incidental catches be reduced? *ICES Journal of Marine Science* 73: 1912-1924

Tyler RM, Targett TE (2007) Juvenile weakfish, *Cynoscion regalis,* distribution in relation to diel-cycling dissolved oxygen in an estuarine tributary. *Marine Ecology Progress Series* 333: 257-269

Wannamaker CM, Rice JA (2000) Effects of hypoxia on movements and behavior of selected estuarine organisms from the southeastern United States. *Journal of Experimental Marine Biology and Ecology* 249: 145-163

Whitney NM, Papastamatiou YP, Holland KN, Lowe CG (2007) Use of an acceleration data logger to measure diel activity patterns in captive whitetip reef sharks, *Triaenodon obesus*. *Aquatic Living Resources* 20: 299-305

Whitney NM, White CF, Gleiss AC, Schwieterman GD, Anderson P, Hueter RE, Skomal GB (2016) A novel method for determining post-release mortality, behavior, and recovery period using acceleration data loggers. *Fisheries Research* 183: 210-221

Zhang H, Ludsin SA, Mason DM, Adamack AT, Brandt SB, Zhang X, Kimmel DG, Roman MR, Boicourt WC (2009) Hypoxia-driven changes in the behavior and spatial distribution of pelagic fish and mesozooplankton in the northern Gulf of Mexico. *Journal of Experimental Marine Biology and Ecology* 381: S80-S91

For Review Only

For Review Only

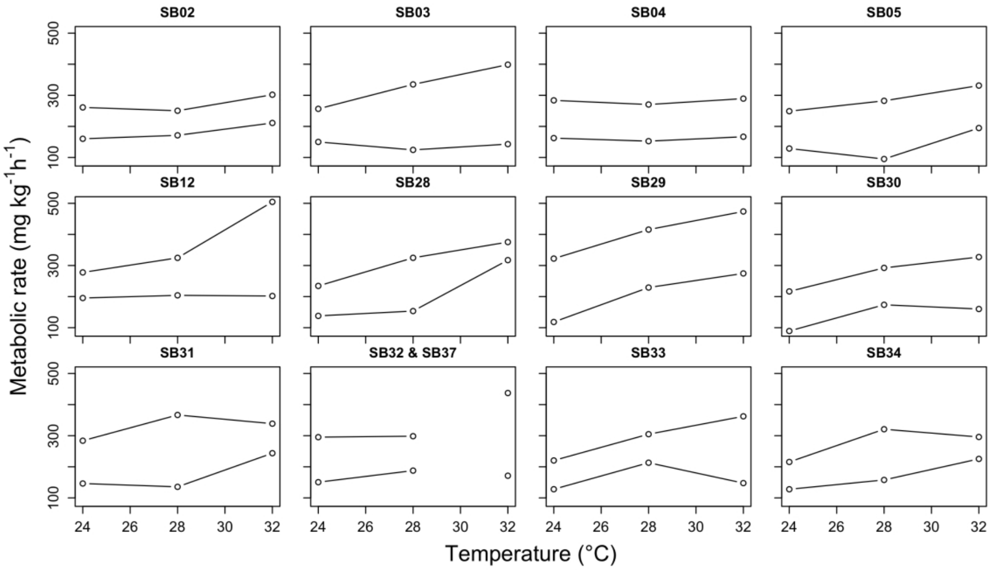

Figure 3

999x583mm (72 x 72 DPI)

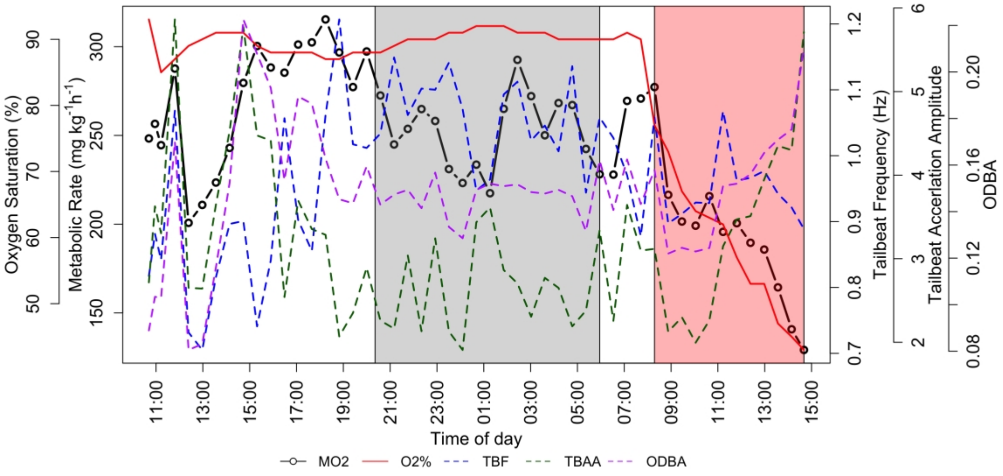

Fig 2

1249x624mm (72 x 72 DPI)

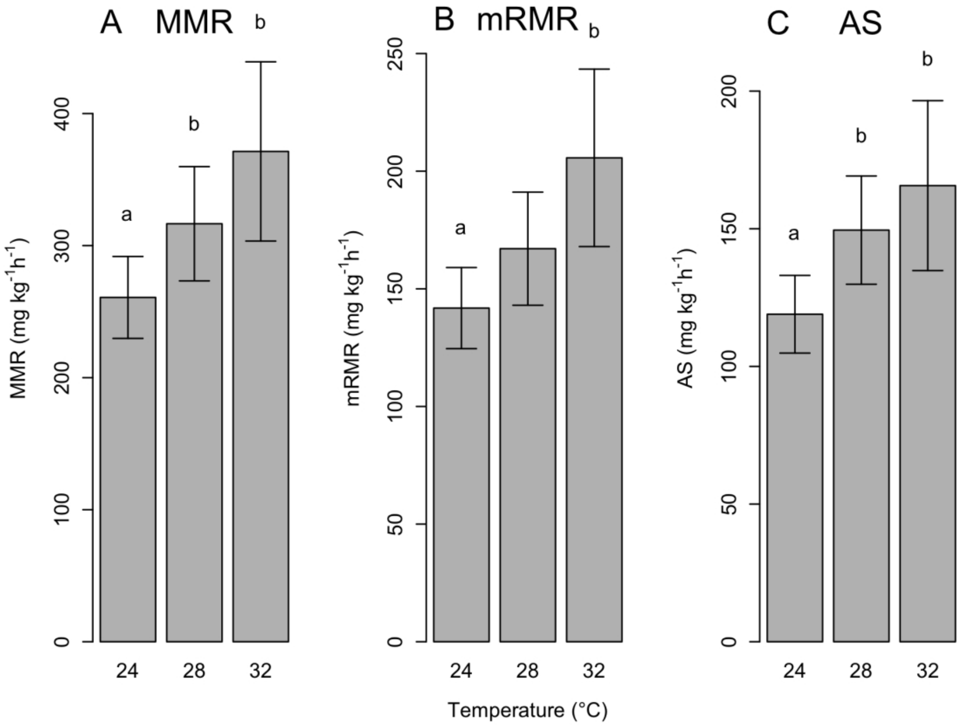

Fig 4

708x583mm (72 x 72 DPI)

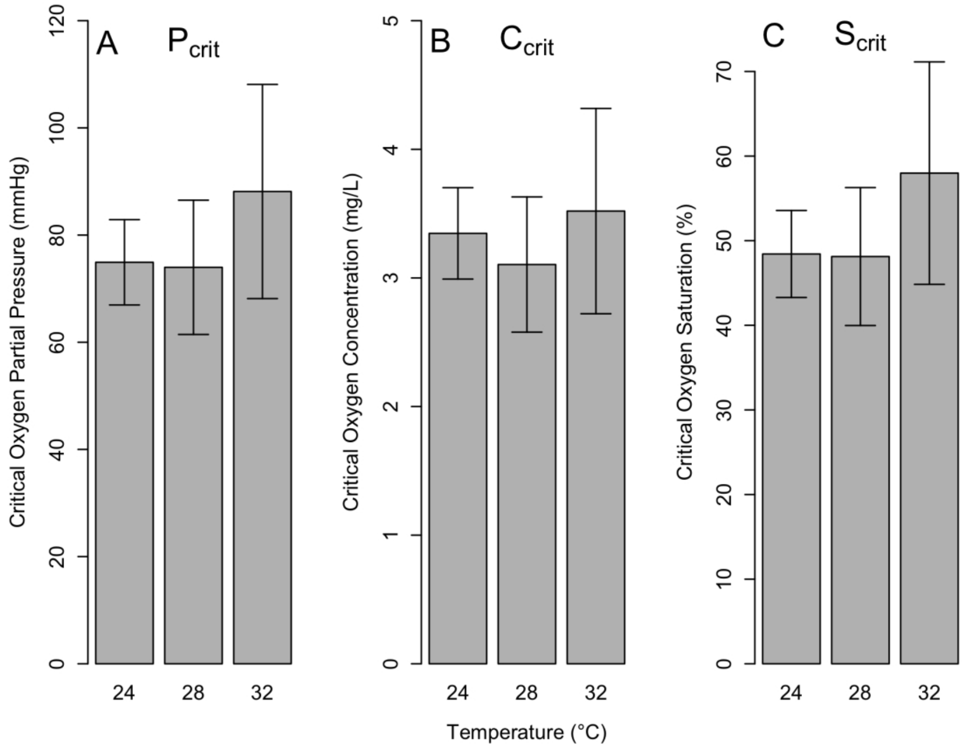

fig 5

708x583mm (72 x 72 DPI)

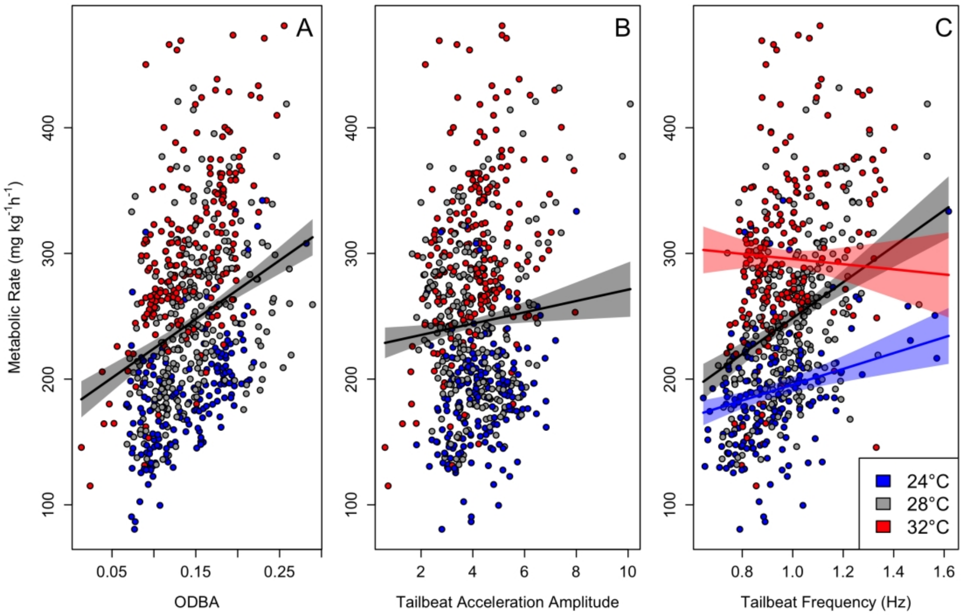

916x624mm (72 x 72 DPI)

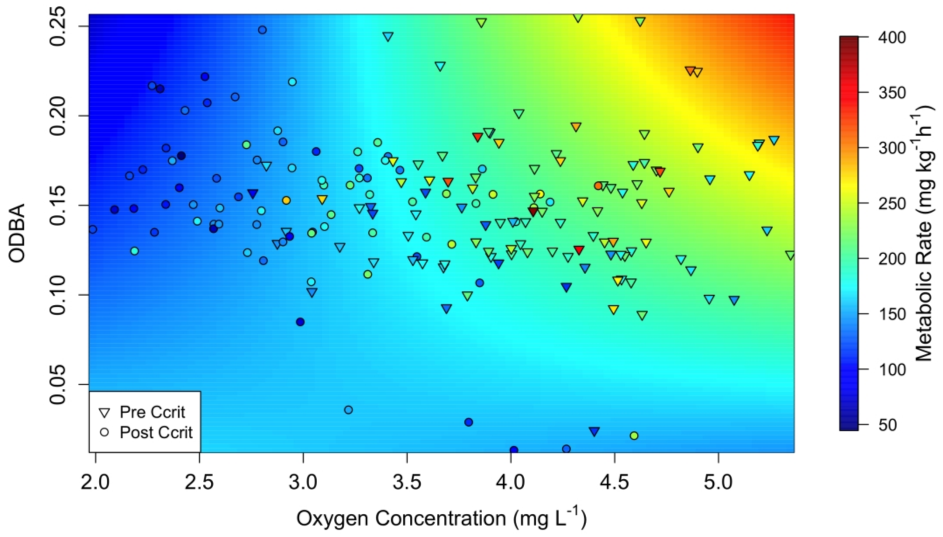

1041x624mm (72 x 72 DPI)
